# Systems Analysis of Transcriptomic and Proteomic Profiles Identifies Novel Regulation of Fibrotic Programs by miRNAs in Pulmonary Fibrosis Fibroblasts

**DOI:** 10.3390/genes9120588

**Published:** 2018-11-29

**Authors:** Steven Mullenbrock, Fei Liu, Suzanne Szak, Xiaoping Hronowski, Benbo Gao, Peter Juhasz, Chao Sun, Mei Liu, Helen McLaughlin, Qiurong Xiao, Carol Feghali-Bostwick, Timothy S. Zheng

**Affiliations:** 1 Biogen, Cambridge, MA 02142, USA; steven.mullenbrock@cellsignal.com (S.M.); fliu@admirxtx.com (F.L.); suzanne.szak@biogen.com (S.S.); xiaoping.hronowski@biogen.com (X.H.); benbo.gao@biogen.com (B.G.); peter.juhasz@biogen.com (P.J.); chao.sun@biogen.com (C.S.); mei.liu@biogen.com (M.L.); helen.mclaughlin@biogen.com (H.M.); qiurong.xiao@biogen.com (Q.X.); 2 Division of Rheumatology and Immunology, Department of Medicine, The Medical University of South Carolina, Charleston, SC 29425, USA; feghalib@musc.edu

**Keywords:** interstitial lung disease, idiopathic pulmonary fibrosis, systemic sclerosis, myofibroblast, gene expression, proteomics

## Abstract

Fibroblasts/myofibroblasts are the key effector cells responsible for excessive extracellular matrix (ECM) deposition and fibrosis progression in both idiopathic pulmonary fibrosis (IPF) and systemic sclerosis (SSc) patient lungs, thus it is critical to understand the transcriptomic and proteomic programs underlying their fibrogenic activity. We conducted the first integrative analysis of the fibrotic programming in these cells at the levels of gene and microRNA (miRNA) expression, as well as deposited ECM protein to gain insights into how fibrotic transcriptional programs culminate in aberrant ECM protein production/deposition. We identified messenger RNA (mRNA), miRNA, and deposited matrisome protein signatures for IPF and SSc fibroblasts obtained from lung transplants using next-generation sequencing and mass spectrometry. SSc and IPF fibroblast transcriptional signatures were remarkably similar, with enrichment of WNT, TGF-β, and ECM genes. miRNA-seq identified differentially regulated miRNAs, including downregulation of miR-29b-3p, miR-138-5p and miR-146b-5p in disease fibroblasts and transfection of their mimics decreased expression of distinct sets of fibrotic signature genes as assessed using a Nanostring fibrosis panel. Finally, proteomic analyses uncovered a distinct “fibrotic” matrisome profile deposited by IPF and SSc fibroblasts compared to controls that highlights the dysregulated ECM production underlying their fibrogenic activities. Our comprehensive analyses of mRNA, miRNA, and matrisome proteomic profiles in IPF and SSc lung fibroblasts revealed robust fibrotic signatures at both the gene and protein expression levels and identified novel fibrogenesis-associated miRNAs whose aberrant downregulation in disease fibroblasts likely contributes to their fibrotic and ECM gene expression.

## 1. Introduction

Interstitial lung disease (ILD) associated with idiopathic pulmonary fibrosis (IPF) and systemic sclerosis (SSc) has a critical impact on a patient’s quality of life and is the predominant cause of mortality in these diseases. Although the pathogenesis of pulmonary fibrosis in IPF and SSc remains incompletely understood, it is generally accepted that they stem from different root causes, with clinical and genetic evidence supporting epithelial injury/dysfunction and vasculopathy/inflammation as the underlying pathogenic triggers for IPF and SSc, respectively [1]. Despite their distinct origins, the resulting persistent tissue injury events converge on pathological fibroblast/myofibroblast activation, culminating in excessive extracellular matrix (ECM) deposition and ultimately progressive loss of lung function in both IPF and SSc [2].

To understand mechanisms underlying pulmonary fibrosis, several groups have undertaken transcriptomic analyses of both tissue and fibroblasts/myofibroblasts derived from fibrotic lung of IPF and SSc patients [3,4,5,6,7,8,9]. These studies found that a limited number of genes, pathways, and functions are altered in pulmonary fibrosis, including TGF-β and WNT, as well as altered expression of ECM genes such as collagens, crosslinking enzymes, TIMPs, and MMPs, many of which have been shown to be functionally relevant in fibrogenesis by subsequent in vitro and in vivo studies. Importantly, key fibrosis-associated pathways (e.g., ECM, WNT, and TGF-β) identified from transcriptomic analyses of fibrotic lung tissue were also captured in the gene signatures of isolated fibroblasts, suggesting that the molecular programming in pulmonary fibrosis is driven in large part by fibroblasts/myofibroblasts.

While these studies have shed insights into mechanisms underlying fibrogenic activation of fibroblasts/myofibroblasts, their fibrotic programs remain incompletely understood. For example, most early studies were performed using microarrays, which are less able to accurately detect low abundance transcripts and are limited to interrogating the expression of the transcripts present on each array platform, which precludes measuring microRNAs (miRNAs) in many cases [3,4,5,6,7,8,9]. To our knowledge, no global miRNA studies have been reported for either IPF or SSc lung fibroblasts and how miRNAs regulate their fibrotic program remains to be characterized. In addition, excessive ECM protein deposition by fibroblasts/myofibroblasts is directly responsible for IPF and SSc disease pathology, yet surprisingly little has been done to characterize the aberrant matrisome protein profile of these disease fibroblasts, which is not only affected by transcriptional changes, but is also subjected to post-transcriptional regulation [10].

To thoroughly interrogate mechanisms by which pathological activation of fibroblasts/myofibroblasts is regulated in IPF and SSc, we performed genome-wide analyses of both messenger RNA (mRNA) and miRNA in these cells, and characterized their ECM deposition properties by proteomic analysis. Altogether, this current study is the first integrative analysis of fibrotic gene and protein signatures, providing novel insights into the multitude of regulatory mechanisms governing the fibrogenic potential of fibroblasts/myofibroblasts in pulmonary fibrosis.

## 2. Materials and Methods

### 2.1. Cell Culture

Primary fibroblasts were isolated from lung tissues of normal donors whose lungs were not used for transplantation and SSc or IPF patients who underwent lung transplantation at the University of Pittsburgh Medical Center under a protocol approved by the institution’s Institutional Review Board. Isolation and subsequent culture of lung fibroblasts was previously described [4].

### 2.2. RNA-seq and miRNA-seq Analysis

RNA-seq libraries were prepared using the Illumina (San Diego, CA, USA) TruSeq RNA Sample kit with poly-T selection and sequenced using a HiSeq (75-bp paired-end reads). Reads were mapped using Tophat (version 2.0.8), transcripts were assembled using Cufflinks (version 2.2.1), and differential expression was calculated using CuffDiff.

miRNA-seq libraries were prepared using the Illumina TruSeq Small RNA Sample Kit and sequenced using an Illumina miSeq (51-bp single-end reads). Bowtie (version 0.12.5) was used to perform a stepwise alignment of fastq files to Illumina databases. Differentially expressed miRNAs were identified using Bioconductor’s limma package.

### 2.3. Real-Time Reverse Transcription-Polymerase Chain Reaction

RNA was reverse transcribed using the High Capacity cDNA Reverse Transcription Kit with RNase inhibitor (Life Technologies/Thermo Fisher Scientific (Waltham, MA, USA) #4374966) or TaqMan MicroRNA Reverse Transcription Kit (Life Technologies/Thermo Fisher Scientific (Waltham, MA, USA #4366596) in conjunction with appropriate miRNA reverse transcription primers (Table S1). Real-time quantitative PCR (see Table S1 for TaqMan probes) was run on a Life Technologies QuantStudio 12K Flex.

### 2.4. Gene Ontology and Signature Analysis

Gene ontology (GO) analysis was conducted using DAVID Bioinformatics Database [11] and gene signature analysis was conducted using NextBio curated studies [12] and pre-ranked gene set enrichment analysis (GSEA) [13]. Ingenuity Pathway Analysis (IPA) was utilized to interrogate pathways upstream of IPF and SSc differentially expressed gene sets.

### 2.5. miRNA Mimic Transfection and Nanostring Gene Expression Analysis

Primary lung fibroblasts were reverse transfected with a miRNA mimic or negative control mimic (2.5 nM final concentration) using Lipofectamine RNAiMAX (Invitrogen/Thermo Fisher Scientific, Waltham, MA, USA) for 48 h, after which gene expression was assayed with the Nanostring (Seattle, WA, USA) platform using a custom codeset of common “fibrosis” genes.

### 2.6. Extracellular matrix Proteomic Profiling

Extracellular matrix proteins deposited by patient-derived fibroblasts after 4 weeks of culture were enriched by a sequential extraction of cellular and extracellular proteins as described previously [14,15]. The extracted soluble and insoluble ECM samples were further processed for proteomic analysis using Thermo Fisher Scientific’s (Waltham, MA, USA) TMT10plex label reagent. The labeled samples were pooled together and fractionated into three fractions. LC-MS of the fractions were acquired using a Thermo Fisher Scientific QE HF mass spectrometer. Peptide identification and quantification were performed using Maxquant [16] searched against the human Swiss-Prot reference database (https://www.uniprot.org/). Protein levels among fibroblast groups were compared by ANOVA test followed by Tukey’s HSD test.

Additional details for Materials and Methods are provided in Supplementary Materials.

## 3. Results

### 3.1. Transcriptional Profiling Identified Similar Dysregulated Gene Expression Programs in Idiopathic Pulmonary Fibrosis and Systemic Sclerosis Lung Fibroblasts

Lung fibroblasts isolated from IPF and SSc patients undergoing lung transplant and unused healthy donor lung were grown under similar culture conditions at low passage number (passage 2-3) prior to performing RNA-seq and miRNA-seq. As reflected by the low forced vital capacity % (FVC) (FVC < 60%) and the patients’ requirement for a transplant, this study provides a snapshot of fibroblasts from patients with severe, end-stage lung disease (Tables S2 and S3).

**Figure 1:**
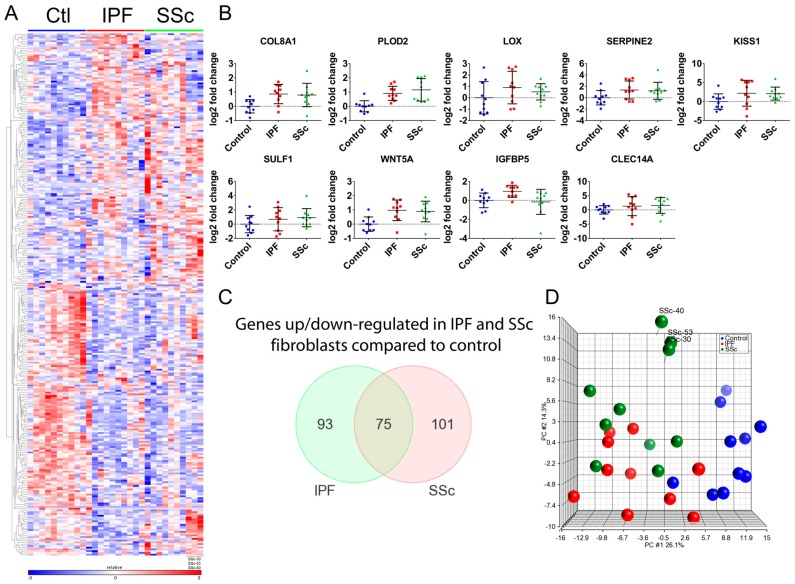
RNA-seq identifies 297 differentially expressed genes (DEGs) across idiopathic pulmonary fibrosis (IPF), systemic sclerosis (SSc), and healthy control primary lung fibroblast comparisons and demonstrates similar disease signatures between IPF and SSc lung fibroblasts. (**A**) Heatmap depicting 297 differentially expressed genes as determined from RNA-seq analysis using Cuffdiff (fold-change ≥+1.5-fold or ≤−1.5-fold, q < 0.05) across all comparisons (IPF vs. Control, SSc vs. Control, SSc vs. IPF). (**B**) Quantitative polymerase chain reaction (qPCR) analysis validated differential expression of several genes involved in profibrotic pathways. Gene expression analysis using real-time qPCR on the 30 patient fibroblast samples was conducted as described in Materials and Methods and normalized to *GAPDH*. Data is plotted as a log2 fold-change relative to the mean of the healthy control samples. (**C**) Venn diagram demonstrating overlap of statistically significant differentially expressed genes between IPF and SSc fibroblasts. (**D**) Principle component analysis on the 30 patient fibroblasts was run using the 297 genes that were significantly different across all patient group comparisons. This allows for the visualization of how similar/different the patient groups are from one another based on disease genes as well as IPF or SSc-specific genes. Healthy control fibroblasts= Ctl.

RNA-seq analysis of the 30 primary lung fibroblasts (n = 10 for each group) identified 297 differentially expressed genes (DEGs) across all cohort comparisons (≥ ±1.5-fold, false discovery rate (FDR) q < 0.05) (Figure 1A and Table S4). We confirmed differential expression of several genes by qPCR (Figure 1B) and expression measured by qPCR and RNA-seq were highly correlated (Figure S1).

The majority of DEGs were observed between disease and control fibroblasts, with 168 DEGs for the IPF vs. Normal and 176 DEGs for the SSc vs. Normal comparison. The IPF and SSc fibroblast gene signatures are very similar, as over 40% of the DEGs overlap between them (*p* < 0.001), and for those that do not overlap their expression changes generally trend in a similar direction (Figure 1A,C). This is further supported by the principle component analysis (PCA) of the patient samples using the 297 DEGs across all comparisons, which illustrates that the IPF and SSc patients cluster close to one another along the first principle component (Figure 1D).

Interestingly we did identify expression differences between IPF and SSc fibroblasts (68 DEGs IPF vs. SSc, ≥ ±1.5-fold, FDR q < 0.05, Figure S2), although this seemed to be driven by a small subset of SSc patients that were among those with notes of pulmonary hypertension, as shown by their clear separation along the second principle component of the PCA plot (Figure 1D), although it should be noted that other SSc patients with pulmonary hypertension did not separate out similarly.

### 3.2. Idiopathic Pulmonary Fibrosis and Systemic Sclerosis Lung Fibroblast Disease Signatures are Associated with Pro-Fibrotic Pathways and Extracellular Matrix

The highly similar gene expression profiles of IPF and SSc lung fibroblasts likely represent a fibrotic disease signature that reflects aberrant activation of upstream signaling that sustains these fibrotic programs and that is directly involved with the pathological function of fibroblasts/myofibroblasts in ILD. Utilizing computational analyses such as GO enrichment analysis, IPA, and gene signature analysis using rank-based directional enrichment tools such as NextBio [12] and GSEA [13], we characterized the IPF and SSc fibroblast signatures to determine how they may relate to both upstream signaling pathways and potential downstream functions.

**Figure 2:**
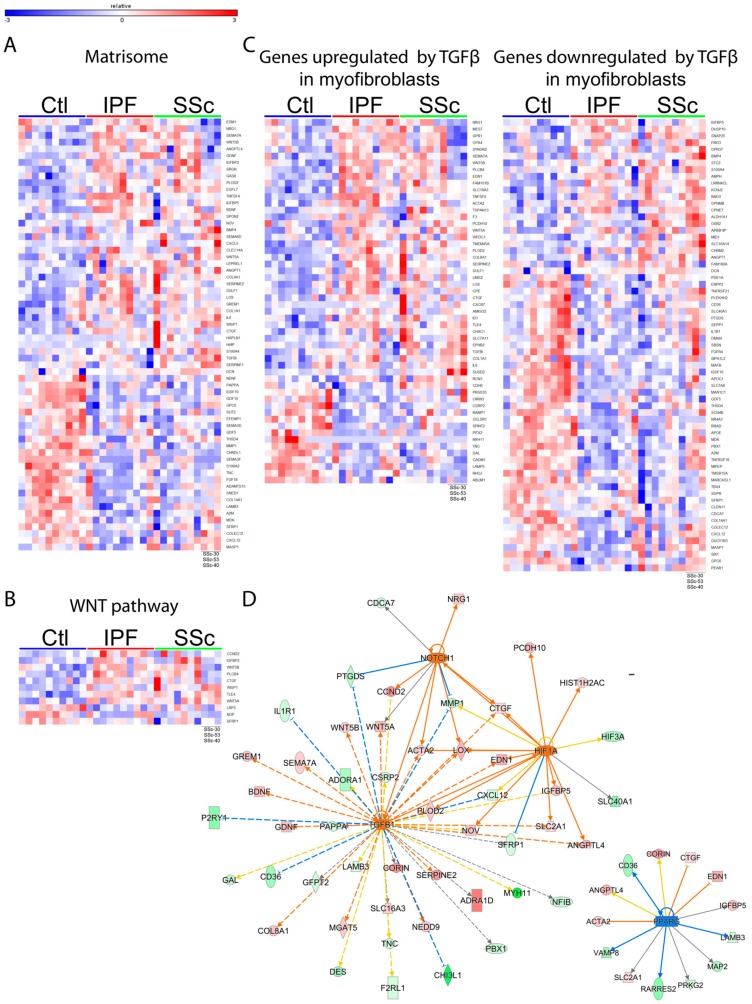
The dysregulated gene expression program in disease fibroblasts is composed of altered matrisome genes and associated with signaling pathways reflective of pathological fibroblast activation. Shown are heatmaps depicting expression of genes differentially expressed in IPF or SSc fibroblasts that overlapped significantly with various gene/pathway signatures such as matrisome (**A**), WNT (**B**), or TGF-β (**C**). (**D**) IPA upstream regulator analysis was used to predict pathway/transcription factor activation state upstream of the IPF or SSc DEGs. The pathways shown here are based on data from IPF DEGs (SSc DEGs had similar results). Shown is the predicted activation/repression of TGF-β, NOTCH1, HIF1A, and PPARG with lines connecting to genes differentially expressed in IPF fibroblasts that are downstream of these pathways. Healthy control fibroblasts= Ctl.

These tools revealed that IPF and SSc fibroblast signatures are associated with the activation of several profibrotic signaling pathways such as WNT (Figure 2B), TGF-β (Figure 2C, Figure S3), NOTCH1 (Figure 2D), and HIF1A (Figure 2D), as well as inhibition of the anti-fibrotic PPARG pathway (Figure 2D).

**Figure 3:**
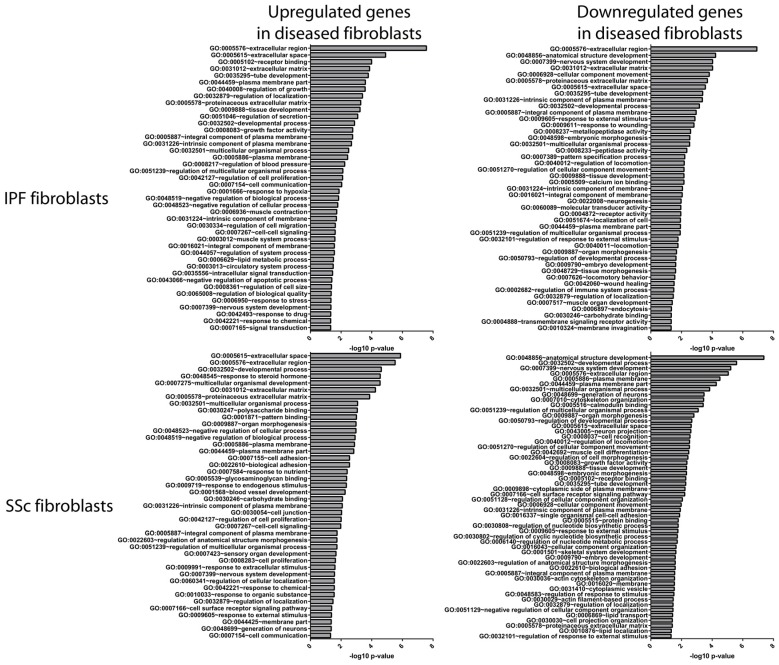
IPF and SSc fibroblast signatures are primarily enriched for genes associated with the extracellular matrix. Gene ontology (GO) analysis was conducted to identify over-represented GO terms for the genes upregulated in IPF or SSc fibroblasts (left panel) and downregulated in IPF or SSc fibroblasts (right panel). Shown are the significantly enriched GO terms (*p* ≤ 0.05, ≥5% of genes had to be classified by a GO term), with highly similar GO terms being collapsed using REVIGO [18].

To further probe potential downstream functions of genes altered in IPF and SSc fibroblasts, we used GO analysis and found that the disease fibroblast gene signatures are enriched for genes associated with “cell proliferation” (IPF and SSc upregulated genes), “muscle contraction” (IPF upregulated genes), “response to wounding” (IPF downregulated genes), and “metallopeptidase activity” (IPF downregulated genes) (Figure 3). In addition, GO terms associated with ECM were the most significant and frequently observed terms for the upregulated and downregulated gene sets for both IPF and SSc. Consistent with this, we also found that the IPF and SSc fibroblast signatures are significantly enriched for components of the in silico matrisome derived by Naba et al. [17], with 64 of the 269 DEGs being associated with the matrisome (Figure 2A, *p* < 0.001).

### 3.3. Global microRNA Profiling Identified Similar Fibrotic microRNA Signatures in Idiopathic Pulmonary Fibrosis and Systemic Sclerosis Lung Fibroblasts

In order to get a more complete picture of the transcriptional profiles of IPF and SSc lung fibroblasts and to identify potentially novel mechanisms by which their expression programs are regulated, we analyzed their miRNA expression using miRNA-seq.

**Figure 4:**
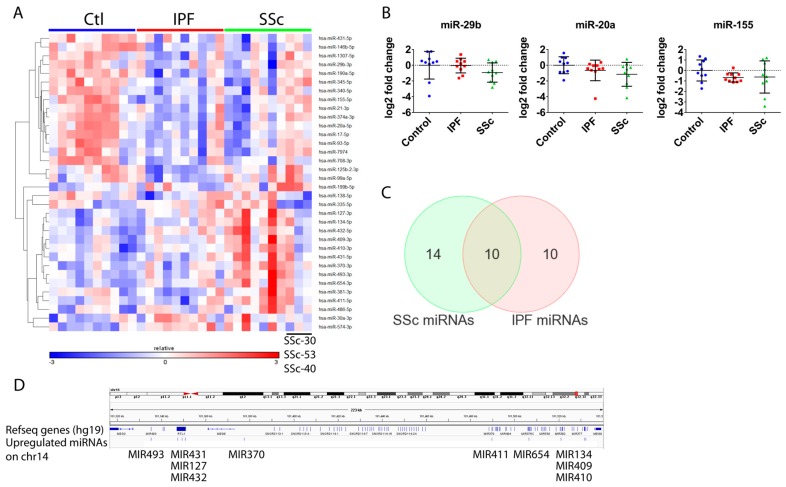
miRNA-seq identified similar miRNA signatures for IPF and SSc lung fibroblasts. (**A**) Heatmap depicting differentially expressed miRNAs across all patient group comparisons (fold-change ≥+1.35-fold or ≤−1.35-fold, *p* < 0.1). (**B**) miRNA expression analysis using real-time qPCR on the 30 patient fibroblast samples was conducted as described in Materials and Methods for miR-20a, miR-155 and miR-29b in order to demonstrate similar expression changes as observed by miRNA-seq. (**C**) Venn diagram depicting overlap between miRNAs that were differentially expressed in SSc and IPF fibroblasts. (**D**) Genome view (hg19) of the Chr14q32 region where a cluster of 10 miRNAs were observed to be upregulated in SSc fibroblasts. Annotated Refseq genes are shown with the location of the indicated upregulated miRNAs marked directly below.

miRNA-seq revealed miRNA expression differences between normal and disease fibroblasts that were relatively moderate (generally <2-fold), which is typical for miRNA expression data. Because of these moderate changes, we utilized a less stringent expression cutoff to minimize the possibility of false negatives. Compared to control fibroblasts, IPF fibroblasts exhibited 3 upregulated and 16 downregulated miRNAs, whereas there were 12 upregulated and 12 downregulated miRNAs for SSc fibroblasts (±1.35-fold, *p* < 0.1, Figure 4A, Table S5). Analogous to the mRNA expression data, the IPF and SSc miRNA signatures were very similar to one another, with ~half of the differentially expressed miRNAs overlapping between them (Figure 4C). Interestingly, as was observed for mRNA expression, there were also minor differences in miRNA expression between SSc and IPF fibroblasts, with 6 miRNAs having higher and 1 miRNA having lower expression in SSc fibroblasts. To increase our confidence in these modest differences observed, we performed a second miRNA-seq and the technical replicate demonstrated high reproducibility of the data, and qPCR analysis further verified differential expression of several miRNAs (Figure 4B).

The resulting miRNA signature contained many dysregulated miRNAs with either established links to fibrosis (e.g., miR-29b and the miR-17~92 cluster), or affecting pathways and processes relevant to IPF and SSc fibroblasts (e.g., TGF-β) (Table S5). Additionally, a number of the miRNAs upregulated in SSc fibroblasts have previously been reported to be upregulated in IPF lung tissue (several miRNAs in the Chr14q32 miRNA cluster, Figure 4D).

### 3.4. Differentially Expressed microRNAs in Idiopathic Pulmonary Fibrosis and Systemic Sclerosis Fibroblasts Regulate Expression of Fibrosis-Associated Genes

To characterize the functional relevance of these dysregulated miRNAs to fibroblast/myofibroblast pathology, we examined whether modulating several miRNAs (miR-29b-3p, miR-138-5p, and miR-146b-5p) in disease fibroblasts would affect expression of ECM and other profibrotic genes. These miRNAs were chosen either because of their extensive associations with fibrotic disease/pathways/ECM (miR-29b-3p) or because their role in fibrosis is relatively uncharacterized, yet they were among the most highly downregulated miRNAs in disease fibroblasts (miR-138-5p, miR-146b-5p).

**Figure 5:**
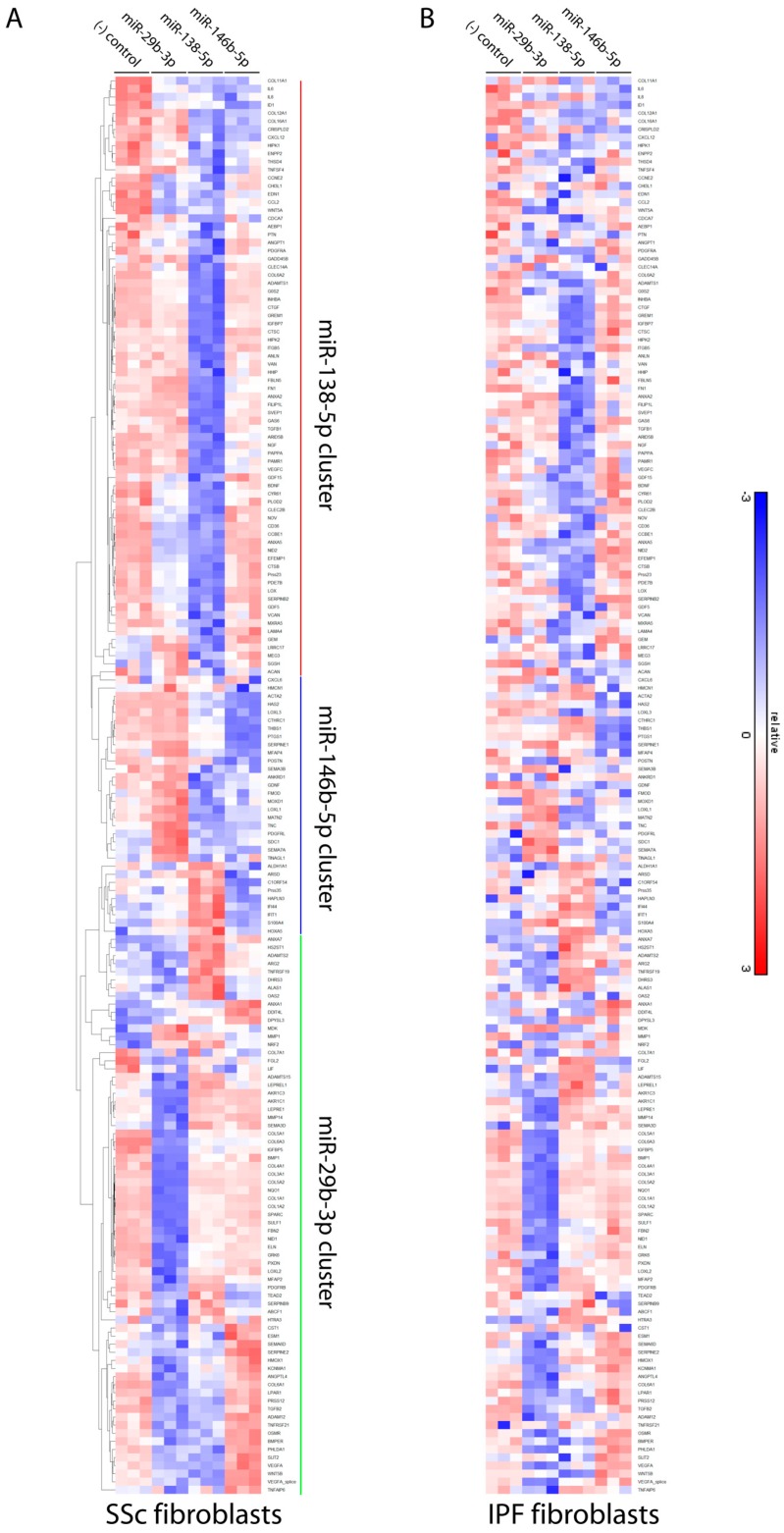
miR-29b-3p, miR-138-5p, and miR-146b-5p mimics downregulate expression of pro-fibrotic genes. miRNA mimics for miR-29b-3p, miR-138-5p, or miR-146b-5p were transfected into primary SSc (**A**) or IPF (**B**) lung fibroblasts as described in Materials and Methods for 48 hours prior to collecting RNA. Gene expression was measured using Nanostring analysis of a panel of 500+ “fibrosis” genes. Shown is a heatmap depicting expression of genes whose expression was significantly affected (≥ +1.5-fold or ≤ −1.5-fold, *p* ≤ 0.05) by at least one of the miRNA mimics in either the SSc or IPF fibroblasts.

Transfection of miR-29b-3p, miR-146b-5p, or miR-138-5p mimics into IPF and SSc lung fibroblasts all had significant effects on ECM and fibrosis gene expression using a “fibrosis” nanostring panel, with 175 genes being modulated by at least one of the miRNAs (Figure 5). As the genes in this panel are generally pro-fibrotic, these miRNA mimics resulted mostly in their downregulation in both IPF and SSc fibroblasts.

As we have gene and miRNA expression from parallel samples, we next aimed to determine if these miRNAs could contribute to the dysregulated fibrotic signature we observed in IPF and SSc fibroblasts. The fibrosis nanostring panel includes 46 genes that were also upregulated in the IPF and SSc fibroblast gene signatures. Of these, 30/46 (65%) were affected by at least one of the miRNA mimics in either SSc or IPF fibroblasts (Figure S4), and in nearly all cases the mimic resulted in downregulation.

Interestingly, each miRNA preferentially affected distinct clusters of genes (Figure 5 and Figure S4). As expected for miR-29b-3p, a number of pro-fibrotic and ECM genes including *COL1A1*, a well-studied target of miR-29, were downregulated. miR-138-5p also reversed expression of a large subset of the disease fibroblast signature, including key pro-fibrotic players such as *LOX*, *CTGF*, and *GREM1*. miR-146b-5p had a more subtle effect on downstream gene expression, however it was the only mimic that significantly reduced *ACTA2* levels in SSc fibroblasts.

### 3.5. Proteomic Profiling Characterized a Distinct Fibrotic Matrisome Deposited by Idiopathic Pulmonary Fibrosis and Systemic Sclerosis Lung Fibroblasts

Aberrant secretion of matrisome and matrisome-associated proteins is both the primary means and end outcome by which fibroblasts drive disease pathology in IPF and SSc ILD. This was reflected in our mRNA profiling data, where ECM/matrisome components were the most prevalent within the fibrotic gene signatures, and in our miRNA-seq data, in which several miRNAs affected matrisome gene expression. As there are additional mechanisms beyond transcriptional regulation that affect ECM production/deposition, we characterized directly the matrisome deposited by IPF, SSc, and normal fibroblasts at the protein level.

Extracellular matrix deposited by the 30 patient fibroblasts (*n* = 10 for each group) was collected and guanidine-soluble and insoluble fractions were subjected to mass spectrometric proteomic profiling separately, as the insoluble fraction is thought to contain more highly cross-linked ECM that could be more relevant in the context of fibrotic disease. In total, 277 matrisome proteins were detected in at least one of the samples. Importantly, using the detected matrisome proteins in the soluble fraction, hierarchical clustering of the fibroblasts resulted in a distinct clustering of IPF and SSc fibroblasts away from controls, strongly suggesting that disease fibroblasts secrete a distinct fibrotic matrisome (Figure S5A). While similar separation was not observed for the insoluble fraction (Figure S5B), the protein recovery for the insoluble fraction was highly variable between different samples, which may have confounded our analysis.

**Figure 6:**
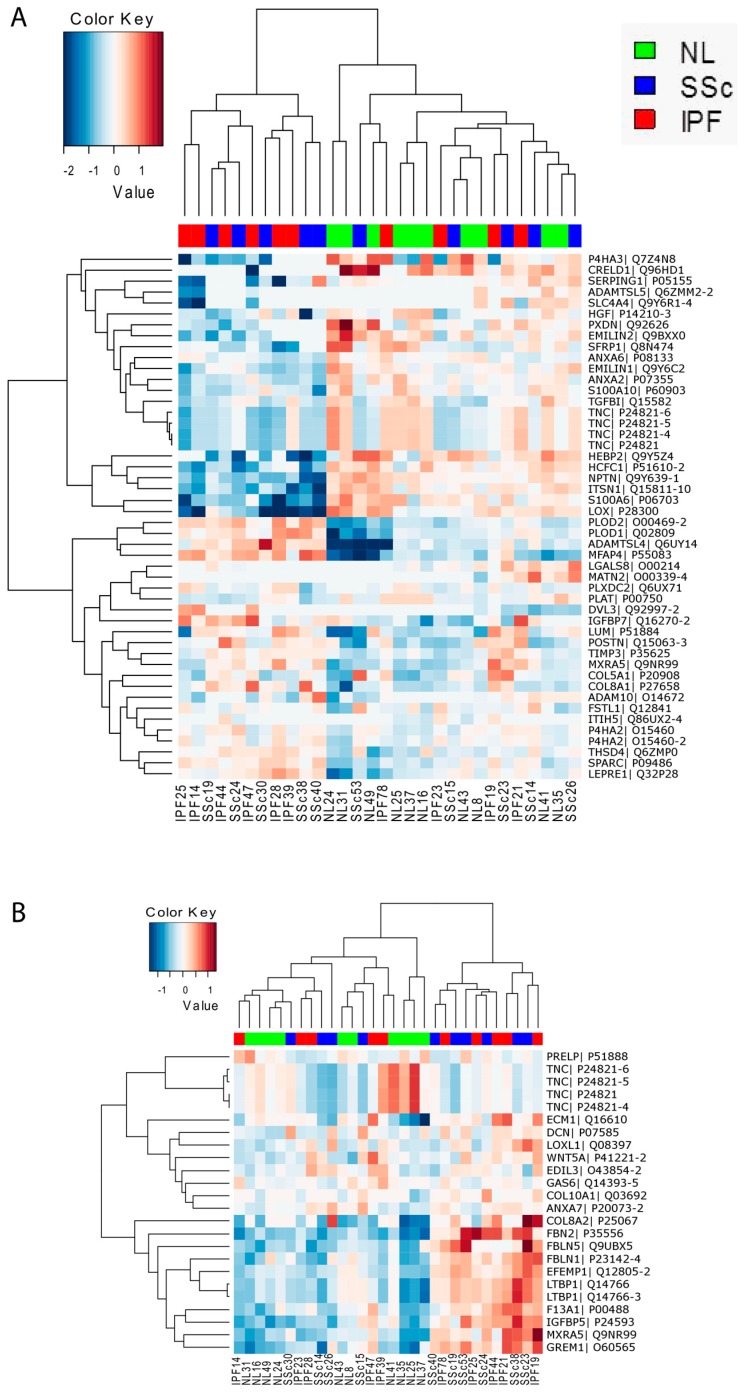
Hierarchical clustering using differentially expressed matrisome proteins is able to delineate normal-like and disease-like fibroblast groups. Shown are heatmaps of z-scored normalized mass spectrometry protein intensity values of matrisome proteins that were differentially expressed across all patient comparisons (fold-change ≥+1.2-fold or ≤−1.2-fold, *p* ≤ 0.05) and then clustered. Shown is the data from the soluble fraction (**A**) and from the insoluble fraction (**B**).

Across all fibroblast group comparisons, the majority of differences were observed between disease vs. normal fibroblasts, with SSc vs. Normal yielding 26 and 18 differentially expressed proteins (DEPs) for the soluble and insoluble fractions respectively and IPF vs. Normal yielding 28 and 6 DEPs for the soluble and insoluble fractions respectively (Figure S5C). Analogous to what we observed at the transcriptional level, the IPF and SSc matrisomes tended to be more similar than different as demonstrated by the observed DEP overlap (Figure S5D) and by the observation that IPF and SSc fibroblasts generally clustered together (Figure S5A and Figure 6). Interestingly we did identify minor differences between SSc and IPF matrisome protein expression, with 8 and 2 DEPs being observed for the soluble and insoluble fractions respectively. Hierarchical clustering using DEPs across all cohort comparisons resulted in a cluster of ~half of the disease fibroblasts when using either the soluble (Figure 6A) or insoluble (Figure 6B) fraction data, demonstrating that disease fibroblasts secrete a distinct matrisome compared to normal fibroblasts.

## 4. Discussion

Fibroblasts/myofibroblasts are key effector cells that directly contribute to the pro-fibrotic milieu, aberrant ECM deposition, increased tissue stiffening, disruption of tissue architecture, and ultimately impaired organ function in pulmonary fibrosis [2]. Understanding the transcriptomic and proteomic profiles of these disease fibroblasts provides insights into their dysregulated fibrotic program at multiple levels and sheds light on potential therapeutic strategies. To our knowledge, the current study is the first attempt at integrating data from mRNA, miRNA, and secreted matrisome of IPF and SSc derived lung fibroblasts and our results revealed a number of novel mechanistic insights into the fibrotic programming of these effector cells underlying pulmonary fibrosis and its complex regulation.

Consistent with previous microarray-based studies, our RNA-seq clearly identified similar “fibrotic gene signatures” for IPF and SSc fibroblasts that reflect a signature of activated myofibroblasts, as illustrated by the correlation for genes upregulated in activated hepatic stellate cells (HSCs) with both the IPF and SSc DEGs (Figure S3). Further computational analysis revealed that these signatures are associated with known fibrotic pathways (WNT, TGF-β, HIF1A, NOTCH1, and PPARG) and effector functions (ECM) of activated fibroblasts/myofibroblasts.

Although the fibrotic transcriptomes from our and previous transcriptomic analyses of fibrotic lung fibroblasts are indicative of pathological myofibroblast activation, our current study also yielded interesting novel findings. First, the exact gene makeup of the reported signatures varied considerably among studies [3,4,5,6,7], which could result from study-specific differences related to patient profiles, source of tissue, culture methods, and profiling methods. However, disease stage is likely a significant driver as we observed stark differences compared to the study reported by Lindahl et al. [6], which used fibroblasts from earlier stage patients, as opposed to end-stage patients in our study. While their study identified a broader set of pathway signatures that were both inflammatory and fibrotic in nature, ECM changes were the most predominant feature in our disease signatures. This suggests that inflammatory changes, such as downregulation of the interferon signature, may be important for progression during early but not at later stages of disease when ECM changes predominate. Second, while previous studies have observed only minimal expression differences between IPF and SSc fibroblasts, our study successfully identified 68 genes that were differentially expressed between them, including inflammation genes (*TNFRSF21*, *CXCL5*, *IL8*) and genes associated with the GO function “oxidoreductase activity” (Figure S2). Interestingly, these differences appear to be driven by a subset of SSc patients (SSc-53, SSc-40, SSc-30). While the precise reason for this is not understood, it could be reflective of concomitant pathological changes frequently associated with SSc, such as pulmonary hypertension and inflammation.

The general similarity between IPF and SSc transcriptomes with subtle differences was also mirrored in the miRNA and matrisome data. Interestingly, for the three SSc patients noted above, we also observed that their miRNA expression tended to differ from the other SSc patients (Figure 4), raising the intriguing possibility that SSc-associated expression changes, such as those that might reflect pathological changes other than fibrosis (e.g., hypertension), could be governed in part at the miRNA level. At the protein level, the secreted matrisome also exhibited a few differences between IPF and SSc. However, unlike the mRNA and miRNA expression data, those three SSc patients did not appear to exhibit distinct matrix protein profiles compared to the other SSc fibroblasts, possibly because the matrisome signature is more reflective of fibrosis and less likely to be indicative of other SSc-associated pathologies. 

As the most salient effector mechanism underlying pulmonary fibrosis, ECM deposition and remodeling by IPF and SSc fibroblasts remain poorly understood. Although the ECM/matrisome transcriptomic signature was the most predominant signal altered in both IPF and SSc fibroblasts, matrix genes were not universally upregulated and in fact many were downregulated (Figure 2A). Thus our data indicate that the fibrotic matrisome does not just result from increased expression of ECM genes but it involves complex dysregulated expression patterns, including both increased and decreased mRNA expression of different collagen types (*COL1A1* upregulation vs. *COL14A1* downregulation), downregulation of ECM-degradation enzymes (*MMP1* and *ADAMTS15*), and upregulation of ECM crosslinking/assembly enzymes (*LOX*, *LEPREL1* and *PLOD2*). Importantly, we confirmed aberrant dysregulation of ECM at the protein level as well by conducting the first proteomic analysis of IPF and SSc fibroblast-deposited matrix. We successfully identified an ECM protein signature shared between IPF and SSc that differentiates disease from healthy fibroblasts, which included several proteins implicated in fibrogenesis such as PLOD2, LUM, POSTN, IGFBP5, GREM1, and SPARC, as well as less characterized ECM proteins such as MXRA5, LEPRE1, MFAP4, and FSTL1. Upon comparing our RNA-seq and mass spectrometry results, we observed similar dysregulation at the protein and mRNA levels for such matrisome components as WNT5A, GREM1, DCN, IGFBP5, COL8A1, PLOD2, SFRP1 and TNC. Surprisingly, these were the only shared dysregulated genes/proteins between the RNA-seq and mass spectrometry data suggesting that differences between protein and mRNA levels of matrisome components are likely due to the presence of a myriad of post-transcriptional regulatory mechanisms at the levels of protein translation, secretion, intracellular and extracellular assembly, as well as enzymatic crosslinking and degradation. However we cannot rule out that the observed differences between the RNA-seq and mass spectrometry results are due to differences in the length of time the fibroblasts were cultured for each experiment, (short-term for RNA-seq, long-term to accumulate sufficient deposited matrix for mass-spectrometry experiments) or that exposure of these cells to tissue culture plastic influenced their protein expression profile.

miRNAs can affect both mRNA levels and protein translation, and a handful of miRNAs have been identified as key players in lung fibrosis. Our current study is the first comprehensive characterization of the global miRNA profiles in fibrotic lung fibroblasts, which included the identification of miRNAs capable of regulating ECM gene expression. Our miRNA-seq analysis identified a number of aberrantly expressed miRNAs in IPF and SSc fibroblasts and several lines of evidence support the relevance of these miRNAs in their fibrotic programming. In particular, the miRNA signature includes many miRNAs previously linked to fibrosis through in vitro and in vivo studies (Table S5). Of particular interest, miR-29b-3p has reduced expression in multiple fibrosis models and human fibrotic disease and can inhibit fibrosis in several mouse models [19,20,21,22,23,24,25,26,27,28,29,30,31,32]. miR-138-5p and miR-146b-5p, which demonstrated some of the highest levels of downregulation in the disease fibroblasts, have been implicated in processes relevant to fibrosis. For example, miR-138-5p has been suggested to play a role in hypertrophic scar fibroblasts during abnormal wound healing [33], osteogenic differentiation and bone formation [34,35], and epithelial-mesenchymal transition [36]. In addition to miR-146b-5p’s effect on TGF-β-signaling [37], its closely-related family member miR-146a inhibits TGF-β-mediated activation of dermal fibroblasts and HSCs, as well as renal fibrosis in the unilateral ureteral obstruction model [38,39,40]. Importantly, we also demonstrated experimentally that several “fibrotic miRNAs” identified in our study (miR-29b-3p, miR-138-5P and miR-146b-5p) regulate the expression of fibrotic/ECM genes. Their identification as important miRNAs modulating distinct aspects of the fibrotic transcriptomes in IPF and SSc lung fibroblasts is highly significant in our view, as this further supports a role for miR-29 as a master ECM regulator, and reveals novel roles for miR-138-5p and miR-146b-5p in pulmonary fibrosis. Since we only measured their effects on transcript levels, our data understates the potential contribution these miRNAs have in the pathology of disease fibroblasts as they could have additional effects on the deposited fibrotic matrisome signature via translation inhibition. It is important to note that these dysregulated miRNAs could be affecting fibrotic/ECM genes either directly or indirectly through other transcriptional regulators.

Equally intriguing is our finding that 10 miRNAs in the Chr14q32 locus appear to be a fibrotic “miRNA module” in SSc fibroblasts, similar to the previously described Chr14q32 region in IPF lung tissue [41]. Additional miRNA clusters, whereby miRNAs/genes exhibited similar differential gene expression and genomic localization, were identified for miR-17-5p and miR-20a-5p, miR-125b-2-3p and miR-99a-5p, and for miR-335-5p and *MEST*. This implies that miRNA modules are dysregulated in IPF and SSc fibroblasts and indeed we see significant expression level correlations for the genes within these clusters (data not shown).

Altogether, through an integrative approach, we successfully characterized distinct mRNA, miRNA, and deposited matrix protein signatures for IPF and SSc fibroblasts, and in doing so identified novel-regulation of fibrotic gene expression by aberrantly expressed miRNAs.

## Supplementary Materials

The following are available online at http://www.mdpi.com/2073-4425/9/12/588/s1, Supplementary Text: Supplementary Materials and Methods, Figure S1: Gene expression values are highly correlative between RNA-seq and qPCR, Figure S2: RNA-seq identifies 68 DEGs between IPF and SSc lung fibroblasts, Figure S3: IPF and SSc fibroblast gene expression profiles reflect activated hepatic stellate cell (HSC) and TGF-β gene signatures, Figure S4: miR-29b-3p, miR-138-5p, and miR-146b-5p mimics downregulate expression of pro-fibrotic genes that are dysregulated in disease fibroblasts, Figure S5: Proteomic profiling of ECM deposited by IPF and SSc lung fibroblasts reveals a fibrotic matrisome signature that delineates them from normal fibroblasts, Table S1: List of Taqman probes and miRNA mimics used, Table S2: Patient information summary, Table S3: Individual patient information, Table S4: RNAseq expression data for DEGs across all cohort comparisons, Table S5: Differentially expressed miRNAs across all patient lung fibroblast comparisons.
