# Supplementary material for: Systems Analysis of Transcriptomic and Proteomic Profiles Identifies Novel Regulation of Fibrotic Programs by miRNAs in Pulmonary Fibrosis Fibroblasts
Source: Genes (Basel). 2018 Nov 29;9(12):588. doi: 10.3390/genes9120588 (PMC6316743; doi:10.3390/genes9120588)
Supplement: Supplementary file 1 [file genes-09-00588-s001.pdf]

Supplementary Materials and Methods

Study population and cell culture

Primary fibroblasts were cultured from lung tissues of patients with SSc or IPF who underwent lung transplantation at the University of Pittsburgh Medical Center under a protocol approved by the institution's IRB. Primary fibroblasts were also cultured form the lung tissues of normal donors whose lungs were not used for transplantation. Culture of lung fibroblasts was previously described [1]. Primary lung fibroblasts were cultured at low passage (passage 2-3 for RNA-seq and miRNA-seq, < passage 7 for additional studies) in Dulbecco's Modified Eagle Medium (DMEM) containing 4.5 g/L glucose, 4 mM L-glutamine, 1 mM sodium pyruvate, 10% fetal bovine serum, penicillin/streptomycin, 0.25 ug/mL Fungizone at 37° C, 5% CO_2.

RNA-seq and miRNA-seq sample preparation and sequencing

Trizol (Life Technologies) was added directly to flasks of primary lung fibroblasts after aspirating culture media. Total RNA was purified as per the manufacturer's protocol, with the addition of an extra chloroform extraction step to ensure adequate removal of phenol from samples. RNA quality was assessed using an Agilent Bioanalyzer. All samples were of good quality (RIN 9.6-10). For RNA-seq experiments (performed at the Broad Institute), library preparation was done using the Illumina TruSeq RNA Sample kit with poly-T selection. Sequencing was performed using a HiSeq with 75-bp paired end reads, with 50 million reads/sample coverage (range 45-90 million reads per sample). For miRNA-seq experiments, library preparation was done using the Illumina TruSeq Small RNA Sample Kit. Sequencing to quantify miRNA levels was done using an Illumina miSeq with 51-bp single-end reads. In general, 97% of miRNA sequencing data were at and above Q30, and 94% of reads passed quality filter. Read numbers/sample ranged from 0.55 million to 2.5 million. For miRNA-seq, technical replicates were run for each sample.

RNA-seq and miRNA-seq data analysis

For RNA-seq analysis, reads were mapped to the human genome build GRCh37 using Tophat (version 2.0.8), and transcripts were assembled using Cufflinks (version 2.2.1). Differential gene expression was calculated using CuffDiff. A gene was considered differentially expressed if it exhibited an average FPKM≥1 in at least one of the cohorts (expressed) and if the fold change was ≥1.5-fold or ≤-1.5-fold, adjusted p-value <0.05. As an additional filter, fold-change based on log2 (FPKM+1) Cufflinks values was used to remove any false positive that resulted from extremely high/low FPKM values observed in a very small subset of samples.

For miRNA-seq analysis, Bowtie (version 0.12.5) was used to perform a stepwise alignment of fastq files to Illumina databases. Reads were first aligned to a database of contaminants; reads which didn't align to contaminants were then aligned to mature miRNA. Read counts for technical replicates were averaged and only miRNAs that averaged at least 100 counts per sample were considered for further analysis; this amounted to 163 miRNAs. Each miRNA's counts were divided by the sum of all miRNA counts for a given sample. This number was multiplied by 100,000 and then the log2 value was taken. The resulting scaled dataset was analyzed to find differentially expressed miRNAs using Bioconductor's limma package. miRNAs were considered differentially expressed if the fold change was ≥+1.35-fold or ≤-1.35-fold, p<0.1.

Heatmaps were generated using GENE-E (Broad Institute) from z-scored log2 (FPKM+1) Cufflinks values (RNA-seq) and from z-scored log2 LIMMA values (miRNA-seq).

Real-time Reverse Transcription-Polymerase Chain Reaction (RT-PCR)

Reverse transcription of RNA samples was carried out using the High Capacity cDNA Reverse Transcription Kit with RNase inhibitor as per the manufacturer's protocol (Life Technologies #4374966). Reverse transcription for miRNA assays was conducted using TaqMan MicroRNA Reverse Transcription Kit (Life Technologies #4366596) in conjunction with appropriate specific miRNA reverse transcription primers for each miRNA (see Table S1). Real-time quantitative PCR reactions consisting of reverse transcription reaction, 2X TaqMan Gene Expression Master Mix (Life Technologies # 4369016), and 20X TaqMan gene expression assays (see Table S1 for list of TaqMan probes used in this study) were run on a Life Technologies QuantStudio 12K Flex according to the manufacturer's protocol. Data was normalized to GAPDH and relative gene expression was calculated using the ΔΔCt method.

Gene ontology and signature analysis of differentially expressed gene sets

Gene ontology (GO) analysis was conducted using DAVID Bioinformatics Database (version 6.7) [2]. In brief, we included enriched GO terms that were associated with ≥ 5% of the gene set and a minimum of 4 genes, $p \leq 0.05$. Redundant GO terms were collapsed using REVIGO and –log10 p-values were plotted for each differentially expressed gene set [3].

Myofibroblast TGF-β (GSE63659) and Hepatic Stellate Cell (HSC) (E-MEXP-2584) signatures were extracted from NextBio curated studies (fold change ≥+1.5-fold or ≤-1.5-fold, $p \leq 0.05$) [4]. A pre-ranked gene set enrichment analysis (GSEA) was performed to determine if these signatures were enriched in the IPF vs. control and SSc vs. control differentially expressed gene lists. IPF and SSc differentially expressed genes were ranked based on their fold-change compared to control patients and then compared to the TGF-β and HSC signatures using GSEA (classic enrichment statistic) [5]. Genes were categorized as being associated with WNT based on KEGG (hsa04310: Wnt signaling pathway) as well as by including additional upstream and downstream components of the pathway based on literature findings

Ingenuity Pathway Analysis (IPA) was utilized to interrogate pathways that may be activated upstream of IPF and SSc differentially expressed gene sets.

miRNA mimic transfection into primary lung fibroblasts

For miRNA mimic transfection studies, primary lung fibroblasts from 2 patients (SSc-15, IPF-19) were reverse transfected with a miRNA mimic or negative control mimic (2.5 nM final concentration) using Lipofectamine RNAiMAX (Invitrogen). In brief, 500 μL of Opti-MEM media, 6.25 μL Lipofectamine RNAiMAX, and a mirVana miRNA or control mimic (Life Technologies, see Table S1) were incubated at room temperature for 20 min. Fibroblasts (3.0×10^5 cells/35 mm^2 well) were then added directly to the transfection mixture and allowed to transfect for 48 hr. After 48 hr, Trizol was directly added to each well and RNA was purified using a Trizol/RNeasy hybrid protocol.

Nanostring sample preparation and data analysis

RNA from miRNA mimic transfection studies was subjected to Nanostring gene expression analysis as per the manufacturer's protocol using a custom codeset consisting of common "fibrosis" genes. The 542 genes in this panel were chosen based on their association with fibrotic disease based on literature and publicly available transcriptional profiling datasets, their association with fibrosis-relevant pathways, and over 400 matrisome components. In brief, the hybridization reaction consisting of 10 μL Reporter CodeSet, 10 μL hybridization buffer, 5 μL sample RNA (100 ng total), and 5 μL Capture ProbeSet was incubated overnight (>12 hr) at 65° C. Hybridized samples were then run and immobilized in a Sample Cartridge using the nCounter Prep Station set to the high sensitivity setting. Data from processed Sample Cartridges were then acquired using the nCounter Digital Analyzer set to high detail (600 fields of view).

Gene expression data was analyzed using nSolver 2.5. Data was normalized using the geometric mean of positive control probes and to the geometric mean of a panel of 15 housekeeping genes. Genes that were low/not expressed (geometric mean of normalized signal ≤10) were excluded, and genes were considered differentially expressed based on fold-change ≥+1.5-fold or ≤-1.5-fold, $p \leq 0.05$.

ECM protein extraction for proteomic profiling

Fibroblasts (n=10 each for IPF, SSc, and control) were cultured for 4-weeks in complete media supplemented with 10 µg/mL ascorbate every 3 days. After 4 weeks of culture, fibroblasts were harvested and the ECM proteins were enriched by a sequential extraction of cellular and extracellular proteins as detailed by Didangelos et al [6] and Decaris et al [7]. Protease/phosphatase inhibitor cocktails and 25mM EDTA were added to the extraction solutions below. Briefly, the cell monolayer was first washed 4 times with cold 1x PBS. Then, 0.5M NaCl, 10 mM Tris-HCl, pH 7.5 was added to the attached monolayer for 2 hours to extract/remove loosely bound proteins. 0.08% SDS was then added and the entire contents of the plate were scraped into a 15-mL tube whereupon it was slowly vortexed overnight to extract cellular proteins. The whole mixture was centrifuged at 16,000 x g and the pellets were collected. The pellets, which were enriched for ECM proteins, were further extracted using 4M guanidine HCl, sodium acetate, pH 5.8 for 48 hours with fast vortex. Following centrifugation (16,000 x g), the supernatants were collected as the guanidine-soluble ECM fraction. The guanidine-insoluble pellet was rinsed 4 times with water and stored at -80° C until further processing. The resulting two ECM-enriched fractions, guanidine-soluble and guanidine-insoluble named "soluble ECM" and "insoluble ECM", were then used for proteomic analysis.

Sample preparation for proteomic analysis

The extracted soluble and insoluble ECM samples were then subjected to further processing for TMT labeled proteomic analysis. Guanidine-insoluble ECM samples were homogenized in 4M urea and 50 mM triethyl ammonium bicarbonate (TEAB) using a GentleMACS apparatus. For the guanidine-soluble ECM samples, protein was extracted with ethanol at -20° C overnight, whereupon the pellet was collected by centrifugation, washed twice with 90% ethanol, air-dried, and resuspended in 8M urea. Protein concentrations were measured using a NanoDrop-1000 spectrophotometer.

20 µg of each sample was reduced by tris(2-carboxyethyl)phosphine (TCEP) at 55° C for 1 hr. The samples were cooled to room temperature and followed by alkylation using iodoacetamide at room temperature for 30 min. Chilled acetone in a volume ratio of 7/1 (7 parts acetone/1 part sample) was added to each sample, which was then stored at -30° C overnight. The samples were centrifuged at 8000 x g, 4° C for 10 minutes and then the samples were inverted to decant the acetone without disturbing the pellet. Pellets were dried for 2-3 minutes and then dissolved in 50 mM triethyl ammonium bicarbonate (TEAB) and 1.6 M urea. The samples were digested with 500 ng trypsin/lys C mix (Promega) at 37° C for 4 hours and then another 500 ng trypsin/lys C mix at 37° C overnight. The resulting peptide pools were then labeled using Thermo Fisher's TMT10plex label reagent according to the manufacturer's protocol. The labeled samples were pooled together and then fractionated into three fractions. LC-MS of the fractions were acquired using a Thermo Fisher Scientific QE HF mass spectrometer, which was equipped with an easy nano-spray source and in-line with a Thermo Easy-nLC 1000 UHPLC. The chromatography separation was performed using an EASY-Spray column, 50cm x 75µm ID, PepMap RSLC C18, 2µm. The mass spectrometer was operated in a positive mode with data-dependent top-10 MS/MS scan mode.

Peptide identification and quantification were performed using Maxquant searched against the human Swiss-Prot reference database. MS/MS spectra searches allowed variable modifications of oxidized methionine, N-terminal acetylation and hydroxyproline. Two steps were used to normalize the raw data: 1) an intra-experimental (within plex) variation step – for each sample, each protein's raw

signal was normalized to the median ion intensity of the protein within the plex, and 2) an inter-experimental (across plexes) variation step – for each sample, each protein's normalized value from step 1) was transformed by dividing it by the mean of all normalized values of the protein obtained in step 1). Protein levels among heathy control, IPF and SSc were compared by ANOVA test followed by Tukey's HSD test. Statistical significance was set at less than 0.05.

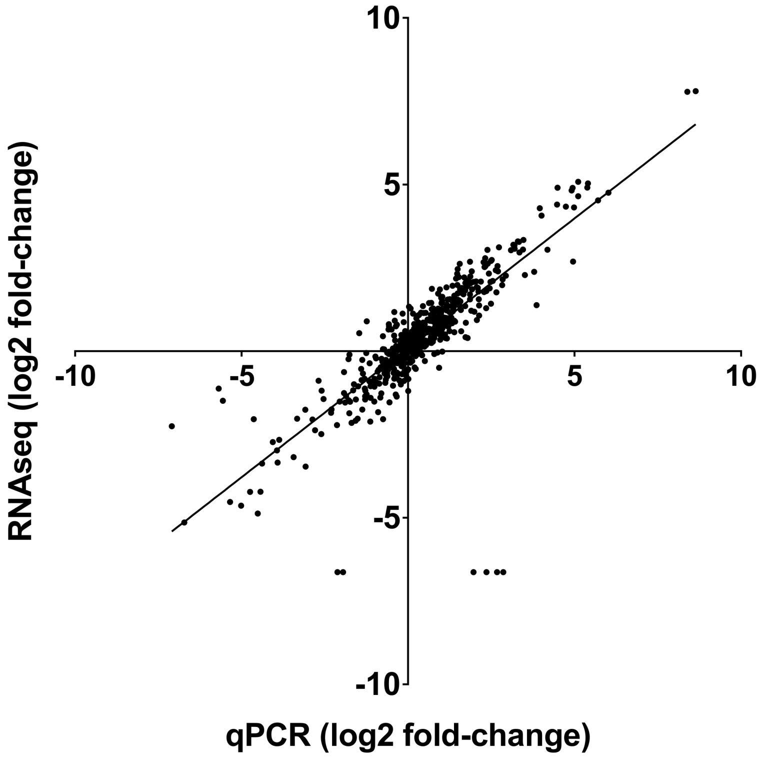

Figure S1. Gene expression values are highly correlative between RNA-seq and qPCR. Expression values for 18 genes across the 30 fibroblast samples were compared across RNA-seq and qPCR. RNA-seq log2 (FPKM+0.01) Cufflinks values were used to generate the log2 fold-change for each gene/sample pair relative to the average control value. These were plotted against the ΔΔCt values obtained from qPCR. Linear regression analysis found a significant correlation for qPCR vs RNA-seq values (R square= 0.6547, $p<0.0001$).

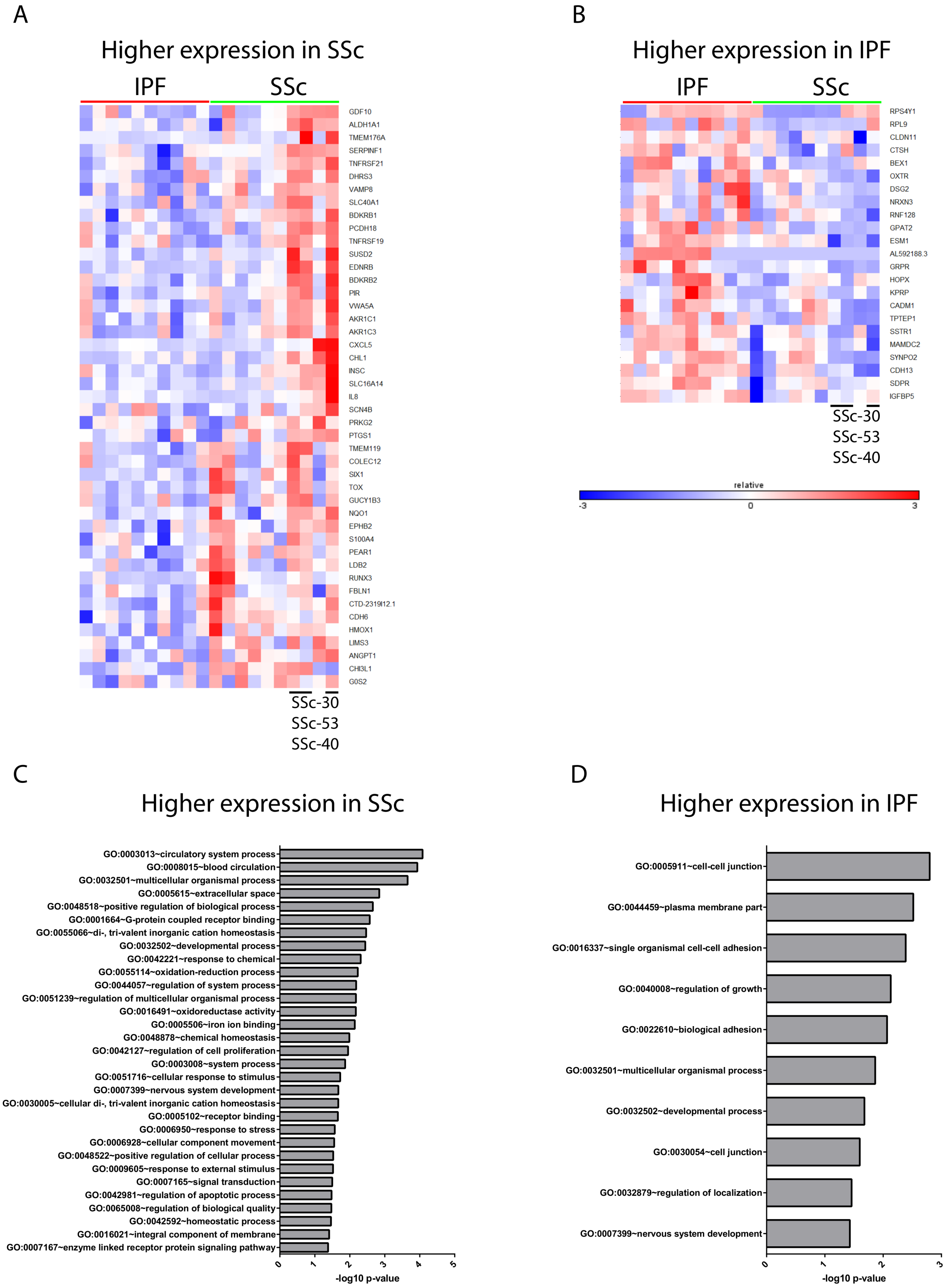

Figure S2. RNA-seq identifies 68 DEGs between IPF and SSc lung fibroblasts. Heatmaps of genes more highly expressed in SSc (A) or in IPF (B) fibroblasts as determined from RNA-seq analysis using Cuffdiff (fold-change ≥+1.5-fold or ≤-1.5-fold, q<0.05) are shown, with the 3 SSc patients that seem to be driving many of the differences indicated below. Gene ontology analysis of the genes more highly expressed in SSc (C) or in IPF (D) fibroblasts was conducted using DAVID. Shown are the significantly enriched GO terms (p≤0.05, ≥5% of genes had to be classified by a GO term), with highly similar GO terms being collapsed using REVIGO.

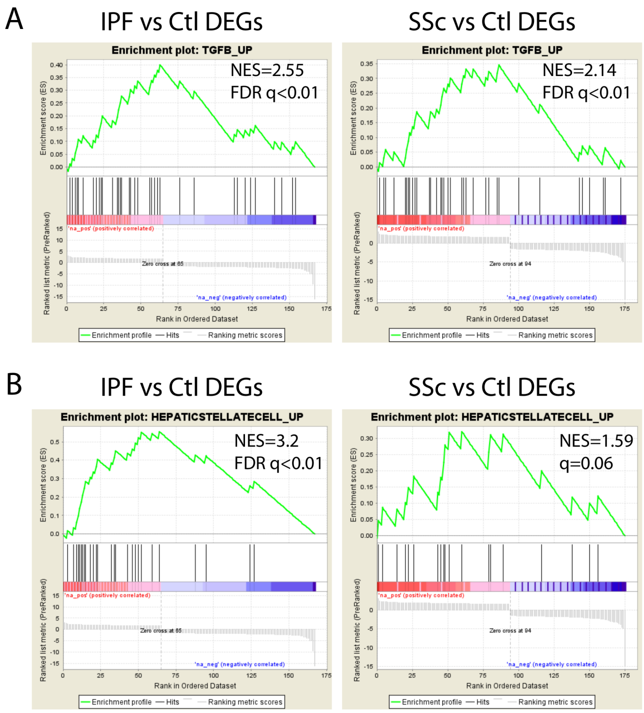

Figure S3. IPF and SSc fibroblast gene expression profiles reflect activated hepatic stellate cell (HSC) and TGF-β gene signatures. A. The TGF-β signature was obtained from NextBio and the upregulated genes were used as a gene set for GSEA analysis on the IPF (left panel) and SSc (right panel) DEGs. B. The activated HSC signature was obtained from NextBio and the upregulated genes were used as a gene set for GSEA analysis on the IPF (left panel) and SSc (right panel) DEGs. Normalized enrichment score (NES) and FDR q values are indicated in the figure.

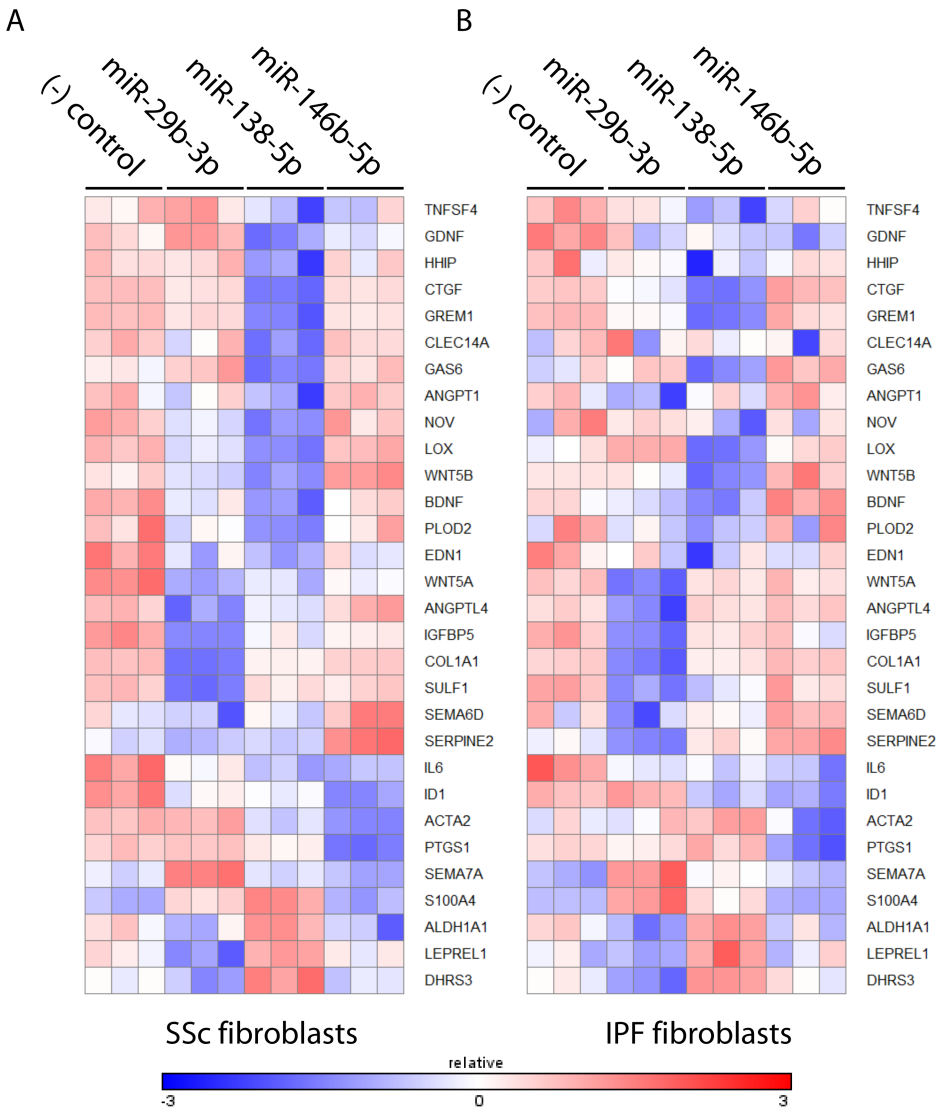

Figure S4. miR-29b-3p, miR-138-5p, and miR-146b-5p mimics downregulate expression of pro-fibrotic genes that are dysregulated in disease fibroblasts. miRNA mimics for miR-29b-3p, miR-138-5p, or miR-146b-5p were transfected into primary SSc (A) or IPF (B) lung fibroblasts as described in Materials and Methods for 48 hours prior to collecting RNA. Gene expression was measured using Nanostring analysis. Shown is a heatmap depicting expression of genes upregulated in disease fibroblasts whose expression was significantly affected (≥+1.5-fold or ≤-1.5-fold, $p \leq 0.05$) by at least one of the miRNA mimics in either the SSc or IPF fibroblasts.

A

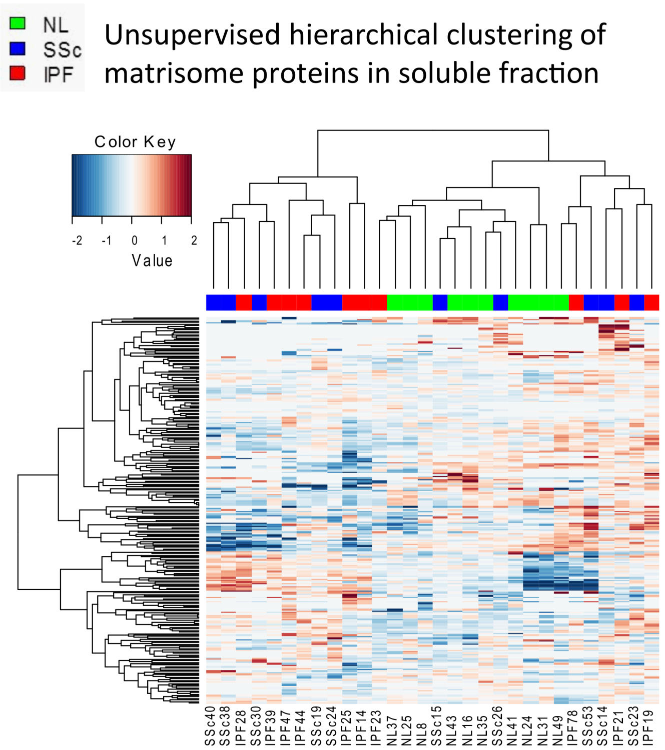

B

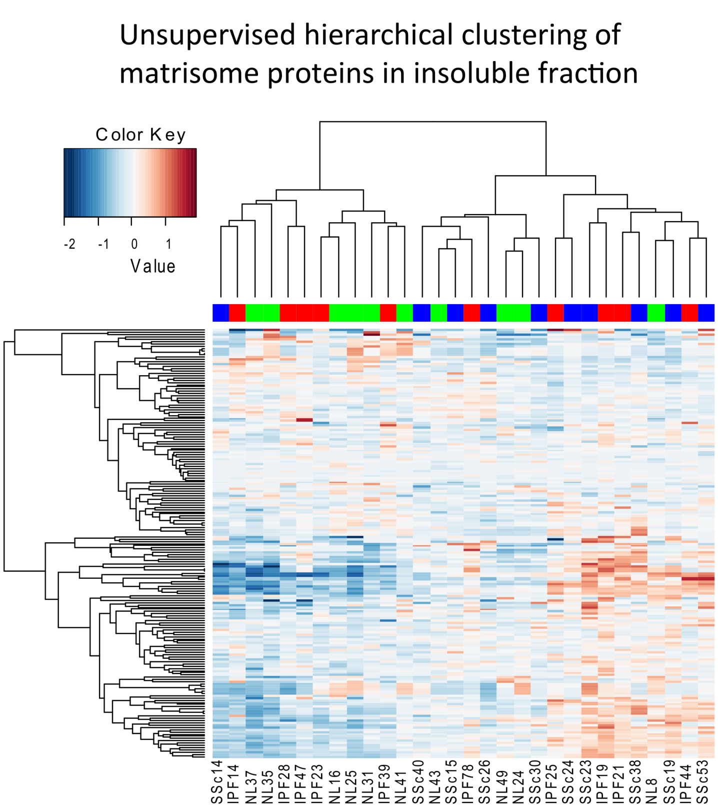

C

	Increased levels		Decreased levels	
	Soluble	Insoluble	Soluble	Insoluble
IPF vs Normal	11	5	17	1
SSc vs Normal	12	14	14	4
SSc vs IPF	5	0	3	2

Fold-change ≥ +/- 1.2-fold, p≤ 0.05

D

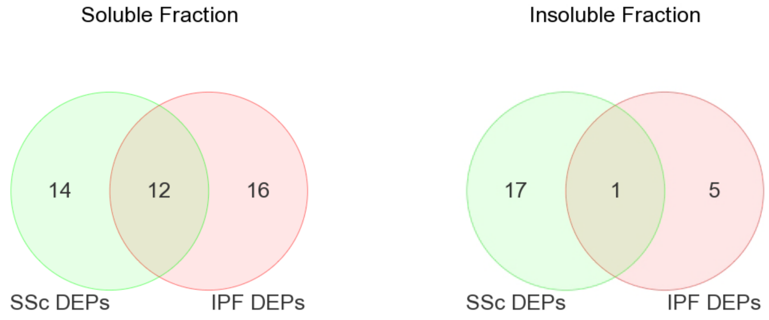

Figure S5. Proteomic profiling of ECM deposited by IPF and SSc lung fibroblasts reveals a fibrotic matrisome signature that delineates them from normal fibroblasts. Mass spectrometric analysis was conducted on ECM secreted by IPF, SSc, or normal fibroblasts after long-term culture. Unsupervised hierarchical clustering using detected matrisome proteins shows 2 distinct groups when utilizing data from the soluble fraction (A) but not when using the insoluble fractions (B). C. Overview of the number of differentially expressed proteins (DEP) between all group comparisons for both the soluble and insoluble fractions. D. Venn diagram illustrating the overlapping DEPs between IPF and SSc fibroblasts for both the soluble and insoluble fractions.

Table S1
List of Taqman probes and miRNA mimics used

**Taqman probes used for qPCR**	
Gene symbol	Taqman Gene Expression Assay ID
ACAN	Hs00153936_m1
ACTA2	Hs00426835_g1
CDH6	Hs01026780_m1
CLEC14A	Hs00545534_s1
COL8A1	Hs00156669_m1
ELN	Hs00355783_m1
FBN2	Hs00266592_m1
GAPDH	4326317E
HAPLN1	Hs00157103_m1
HEATR6	Hs00375382_m1
IGFBP5	Hs00181213_m1
KISS1	Hs00158486_m1
LOX	Hs00942480_m1
LOXL2	Hs00158757_m1
PLOD2	Hs01118190_m1
PXDN	Hs00395488_m1
SERPINE2	Hs00385730_m1
SULF1	Hs00290918_m1
WNT5A	Hs00998537_m1
**Taqman miRNA assays**	
miRNA	miRNA assay ID
hsa-miR-29b	000413
hsa-miR-20a	000580
hsa-miR-155	002623
**miRNA mimics (mirVana)**	
miRNA	mirVana miRNA mimic ID
hsa-miR-146b-5p	MC10105
hsa-miR-138-5p	MC11727
hsa-miR-29b-3P	MC10103
mirVana miRNA Mimic, Negative Control #1	4464058

Table S2
Patient information summary

	Normal n=10	IPF n=10	SSc n=10
Mean age (years)	48.8	60.9	47.2
Gender			
Male	4	8	4
Female	6	2	6
Smoking history			
yes	5	6	4
no	2	4	6
n/a	3	0	0
SSc skin disease			
Limited			3
Diffuse			4
n/a			3
Additional parameters mean ±SD			
FVC %	n/a	43.1±8.9	31.6±10.6
DLCO %	n/a	31.3±13.6	23.3±6.0
PA mean (mmHg)	n/a	25.2±6.7	22.6±4.9

Table S3
Individual patient information

Sample ID	Patient Group	Sex	Age (years)	FVC %	DLCO %	PA Mean (mmHg)	Smoking	Skin Disease Type
NL-16	Control	Female	49				+smoke	
NL-24	Control	Female	44				+smoke	
NL-25	Control	Female	39				+smoke	
NL-31	Control	Female	75				-smoke	
NL-41	Control	Female	53				+smoke	
NL-49	Control	Female	24				-smoke	
NL-35	Control	Male	56				n/a	
NL-37	Control	Male	50				n/a	
NL-43	Control	Male	44				+smoke	
NL-8	Control	Male	54				n/a	
IPF-21	IPF	Female	53.8	39	17	35	-smoke	
IPF-39	IPF	Female	53.7	40	19	19	-smoke	
IPF-14	IPF	Male	46.3	33	29	34	+smoke	
IPF-19	IPF	Male	58.4	55	30	19	-smoke	
IPF-23	IPF	Male	63.9	41	61	23	+smoke	
IPF-25	IPF	Male	66.6	58	35	28	+smoke	
IPF-28	IPF	Male	59.8	46	42	16	-smoke	
IPF-44	IPF	Male	63.8	50	22	29	+smoke	
IPF-47	IPF	Male	68	36	-	20	+smoke	
IPF-78	IPF	Male	75.1	33	27	29	+smoke	
SSc-14	SSc	Female	34.6	28	32	-	-smoke	Diffuse
SSc-15	SSc	Female	47.7	18	-	14	+smoke	Diffuse
SSc-19	SSc	Female	37	18	-	26	-smoke	Diffuse
SSc-23	SSc	Female	51.7	36	20	22	+smoke	Limited
SSc-30	SSc	Female	58.8	39	14	19	-smoke	n/a
SSc-38	SSc	Female	49	26	19	21	+smoke	Limited
SSc-24	SSc	Male	45.1	26	30	23	-smoke	n/a
SSc-26	SSc	Male	57.5	33	26	24	-smoke	Diffuse
SSc-40	SSc	Male	44.3	40	24	32	-smoke	Limited
SSc-53	SSc	Male	46.4	52	21	22	+smoke	n/a

Table S4

RNAseq expression data for DEGs across all cohort comparisons (IPF vs Normal, SSc vs Normal, SSc vs IPF)

IPF vs Normal DEGs

		SSc vs Normal		IPF vs Normal		SSc vs IPF	
gene	locus	log2(fold_change)	q_value	log2(fold_change)	q_value	log2(fold_change)	q_value
LCE3A	1:152595083-152596370	0.260375	0.999971	3.39435	0.00302503	-3.13397	0.00588648
KISS1	1:204159468-204165614	1.76444	0.0276966	2.93945	0.00302503	-1.17501	0.0544352
CLEC14A	14:38720656-38747180	2.66734	0.00302503	2.81147	0.00302503	-0.144133	0.999971
ADRA1D	20:4201236-4230191	1.45214	0.0112285	2.2127	0.00302503	-0.760564	0.382056
AL589743.1,RP11-146E13.2,RP11-496I2.4	14:19650017-19925346	2.29558	0.00723681	2.17153	0.0137916	0.124054	0.999971
CCND2	12:4357930-4414516	1.49718	0.00302503	2.01021	0.00302503	-0.513032	0.568482
GPAT2	2:96676307-96705199	-0.176993	0.999971	1.89291	0.0137916	-2.0699	0.0150479
CORIN	4:47595959-47840385	1.063	0.034065	1.88389	0.00302503	-0.820891	0.0276966
SERPINE2	2:224839828-224904036	2.13461	0.00302503	1.87439	0.00302503	0.260226	0.999971
AP000525.8	22:16147975-16193072	1.82934	0.00302503	1.82142	0.00302503	0.0079158	0.999971
BEX1	X:102317578-102319168	0.317879	0.999971	1.70896	0.00723681	-1.39108	0.00856372
FMO3	1:171060010-171087008	1.7533	0.00302503	1.64992	0.00302503	0.103374	0.999971
PLCB4	20:9049351-9461889	1.50913	0.00302503	1.64647	0.00302503	-0.13734	0.999971
WFDC1	16:84328229-84363457	1.51177	0.00302503	1.42874	0.00302503	0.0830339	0.999971
EDN1	6:12290328-12297427	0.966784	0.0700105	1.39791	0.00302503	-0.431125	0.938352
ANGPTL4	19:8428172-8469318	1.16155	0.329233	1.34145	0.00989026	-0.179906	0.999971
LOX	5:121297655-121414246	0.870875	0.00302503	1.31352	0.00302503	-0.442642	0.642502
WNT5A	3:55499740-55523973	1.17421	0.00302503	1.24029	0.00302503	-0.0660868	0.999971
AMIGO2	12:47469489-47630443	1.82472	0.00302503	1.2227	0.00302503	0.602022	0.2818
BDNF	11:27528384-27899195	1.02968	0.142	1.20237	0.0276966	-0.172689	0.999971
TNFSF4	1:173152803-173446318	0.520467	0.946835	1.18406	0.0150479	-0.663596	0.524989
MEST	7:130126045-130148506	1.04211	0.198317	1.16044	0.00723681	-0.118337	0.999971
PCDH10	4:134070461-134129589	0.831622	0.0575555	1.15171	0.00302503	-0.320086	0.999971
PSG1,PSG7,PSG8	19:43256837-43441330	1.4418	0.00302503	1.14606	0.0150479	0.295732	0.999971
SSTR1	14:38675857-38682272	0.0621314	0.999971	1.14106	0.00302503	-1.07893	0.00302503
LIMS2	2:128395955-128439360	0.952511	0.0124936	1.13451	0.00302503	-0.182004	0.999971
GDNF	5:37812778-37876051	0.983379	0.0436908	1.13282	0.0330131	-0.149444	0.999971
SULF1	8:70378858-70573150	1.49896	0.00302503	1.08998	0.00388754	0.408984	0.896417
CPNE7	16:89642175-89663654	1.04916	0.00302503	1.08782	0.00302503	-0.0386525	0.999971
GPR37	7:124384367-124406040	1.30867	0.00302503	1.06721	0.00989026	0.241457	0.999971
IGFBP5	2:217536827-217858722	0.236752	0.999971	1.05428	0.00302503	-0.81753	0.00302503
COL8A1	3:99357298-99518070	1.04466	0.00302503	1.04585	0.00302503	-0.00119162	0.999971
SLC8A1	2:40013592-40838193	0.587092	0.159931	1.04428	0.00302503	-0.457193	0.402752

KCNS3	2:18059113-18542882	0.493573	0.718216	1.02885	0.00588648	-0.53528	0.478713
NRG1	8:31496901-32627722	0.549885	0.672092	1.0256	0.0162705	-0.475712	0.764781
SERINC2	1:31882411-31907525	0.663769	0.00302503	1.02403	0.00302503	-0.360261	0.347042
NOV	8:120428545-120474590	0.608123	0.0436908	0.990075	0.00302503	-0.381952	0.576396
GREM1	15:33009470-33026870	0.666699	0.132873	0.970882	0.00302503	-0.304183	0.999971
PLOD2	3:145777312-145881440	1.08195	0.00302503	0.966321	0.00302503	0.115627	0.999971
CPA4	7:129932973-129964020	0.669192	0.0750133	0.965885	0.00302503	-0.296693	0.997181
TMEM47	X:34645180-34675405	0.690343	0.00302503	0.947849	0.00302503	-0.257506	0.758721
MGAT5	2:134877512-135212305	0.66778	0.00469276	0.942995	0.00302503	-0.275215	0.846248
FAM70B	13:114462168-114517784	0.906349	0.059618	0.936463	0.0362438	-0.030114	0.999971
NEDD9	6:11173684-11382581	0.895117	0.00302503	0.936184	0.00302503	-0.0410672	0.999971
ACTA2	10:90639490-90775542	0.243406	0.999971	0.910766	0.025545	-0.667361	0.161756
C5orf30	5:102594402-102614361	0.91983	0.00302503	0.907649	0.00302503	0.0121806	0.999971
RAB3B	1:52373624-52456436	0.569176	0.0426246	0.891563	0.00302503	-0.322387	0.733166
SLC16A3	17:80186292-80231607	0.83945	0.00302503	0.861532	0.00302503	-0.0220827	0.999971
CPE	4:166282345-166419472	0.704178	0.0870136	0.849301	0.0187202	-0.145123	0.999971
GPR1	2:207040000-207082771	0.363936	0.895446	0.830978	0.00856372	-0.467041	0.43011
WNT5B	12:1639056-1760860	0.558916	0.025545	0.8096	0.00302503	-0.250684	0.882927
SEMA7A	15:74701629-74726808	0.65627	0.0124936	0.808092	0.00388754	-0.151822	0.999971
FAM101B	17:289768-295730	0.851016	0.00302503	0.802211	0.00302503	0.0488044	0.999971
DUSP10	1:221874765-221915518	0.68783	0.00469276	0.797301	0.00302503	-0.109471	0.999971
CHN1	2:175664090-175870106	0.837281	0.00302503	0.79232	0.00302503	0.0449605	0.999971
HIST1H2AC	6:26124329-26139338	0.878435	0.00302503	0.763555	0.00388754	0.11488	0.999971
ATP8B1	18:55297533-55470669	0.521838	0.0221778	0.750932	0.00302503	-0.229095	0.924027
AC092535.1,SPON2	4:1155624-1202750	0.76429	0.00302503	0.732879	0.00302503	0.0314109	0.999971
SRGN	10:70847861-70864567	1.06947	0.00302503	0.710457	0.00302503	0.359012	0.304714
CTGF	6:132269093-132398533	0.780186	0.00302503	0.708514	0.00302503	0.0716713	0.999971
SLC2A1	1:43391518-43449029	0.847984	0.00302503	0.699794	0.00302503	0.14819	0.999971
FAM180A,SLC13A4	7:135347237-135433892	0.527076	0.30166	0.675361	0.034065	-0.148284	0.999971
SYNGR2	17:76164638-76169608	0.559847	0.13198	0.647745	0.0266209	-0.0878978	0.999971
GAS6	13:114518602-114570155	0.461723	0.126443	0.644653	0.00469276	-0.18293	0.999971
CLGN	4:141309520-141349122	-0.556058	0.0730224	-0.59764	0.0319506	0.0415824	0.999971
MDGA1	6:37598454-37667082	-0.735376	0.00388754	-0.627497	0.0124936	-0.107878	0.999971
GALNT6	12:51745680-51902980	-0.704886	0.00302503	-0.632552	0.00723681	-0.0723339	0.999971
GFPT2	5:179727676-179780387	-0.29853	0.896417	-0.642884	0.0276966	0.344354	0.801914
EFEMP1	2:56093101-56151274	-0.743544	0.00302503	-0.64853	0.00302503	-0.095014	0.999971
F2RL1	5:76114680-76131214	-0.996228	0.00302503	-0.675349	0.025545	-0.320878	0.961697
SLIT2	4:20253351-20623803	-0.741785	0.00723681	-0.6899	0.0150479	-0.0518853	0.999971
CPD	17:28705915-28797007	-0.513292	0.0175003	-0.692151	0.00302503	0.178859	0.999971

SFRP1	8:41119480-41167016	-0.795026	0.00302503	-0.694752	0.00723681	-0.100273	0.999971
PEAR1	1:156863148-156886226	0.532751	0.144725	-0.697383	0.0319506	1.23013	0.00302503
TNFRSF21	6:47199267-47277735	0.228251	0.999971	-0.70709	0.0298268	0.935342	0.00302503
LAMB3	1:209788219-209825811	-0.356518	0.753179	-0.729435	0.0175003	0.372917	0.786035
PAPPA	9:118916050-119164601	-0.632865	0.0586144	-0.738314	0.00989026	0.105449	0.999971
OSBPL8	12:76745310-76953589	-0.913699	0.00856372	-0.748986	0.0276966	-0.164713	0.999971
PRDM6	5:122421426-122530273	-0.328545	0.896417	-0.754989	0.0112285	0.426444	0.646468
EVL	14:100437756-100610653	-0.412054	0.485862	-0.757724	0.00856372	0.34567	0.780569
CXCL12	10:44793037-44881941	-0.238629	0.999971	-0.770174	0.00302503	0.531545	0.0831223
LRP5	11:68080076-68216743	-0.329383	0.661305	-0.771227	0.00302503	0.441844	0.267251
THSD4	15:71389290-72075722	-1.02987	0.00302503	-0.779248	0.00302503	-0.250621	0.999971
MIPEP	13:24304327-24476794	-0.298163	0.871243	-0.801992	0.00302503	0.503829	0.166874
CDCA7	2:174202946-174233725	-0.841555	0.0298268	-0.820206	0.0298268	-0.0213485	0.999971
TMEM119	12:108983621-108992096	0.50707	0.0890229	-0.824454	0.00302503	1.33152	0.00302503
IL1R1	2:102681003-102798568	-0.274836	0.828096	-0.824874	0.00302503	0.550038	0.0394198
PLEKHH2	2:43864398-43995204	-0.420501	0.600555	-0.828918	0.00989026	0.408417	0.733914
GUCY1B3	4:156680032-156728746	0.0368626	0.999971	-0.831847	0.0426246	0.86871	0.0372946
CSRP2	12:77252002-77274132	-0.89494	0.00302503	-0.835992	0.00302503	-0.0589481	0.999971
GPC6	13:93878879-95060937	-0.298366	0.629441	-0.844849	0.00302503	0.546483	0.0351647
TNC	9:117782805-117880765	-1.21006	0.00302503	-0.861244	0.00388754	-0.348821	0.844404
MAOB	X:43625772-43741693	-1.13845	0.00302503	-0.87574	0.00469276	-0.262706	0.999971
PDE1A	2:183004595-183387919	-0.56736	0.0955995	-0.894845	0.00302503	0.327485	0.910801
MDK	11:46402305-46405375	-0.470103	0.140139	-0.902118	0.00302503	0.432015	0.34256
DCHS1	11:6642555-6677573	-1.13556	0.00302503	-0.905467	0.00302503	-0.23009	0.999971
MAMSTR,RASIP1	19:49210641-49243978	-0.127834	0.999971	-0.935168	0.0266209	0.807334	0.0870136
PRKG2	4:82007662-82137627	0.0254716	0.999971	-0.940984	0.00302503	0.966456	0.0162705
NFIB	9:14081841-14398982	-0.949975	0.00302503	-0.947899	0.00302503	-0.00207633	0.999971
FAM213A	10:82167584-82195320	-1.47801	0.00302503	-0.959602	0.030883	-0.518405	0.852231
SBSN	19:36014268-36019317	-0.44975	0.955748	-0.979699	0.0351647	0.529949	0.871243
S100A2	1:153533583-153540366	-0.98818	0.0533991	-0.989821	0.0416127	0.00164172	0.999971
ENPP2	8:120569323-120685693	-0.793009	0.00302503	-0.992212	0.00302503	0.199204	0.999971
PBX1	1:164524820-164868533	-0.837945	0.00469276	-0.999217	0.00302503	0.161272	0.999971
GAL	11:68451246-68458645	-1.30872	0.116091	-1.01237	0.0426246	-0.296346	0.999971
RAMP1	2:238767535-238820756	-0.376645	0.896417	-1.01625	0.00302503	0.639607	0.282389
ADAMTS15	11:130317906-130346541	-1.07791	0.00302503	-1.06821	0.00302503	-0.00969856	0.999971
LAMP5	20:9485826-9511171	-2.54364	0.00302503	-1.09282	0.00302503	-1.45083	0.00302503
MAFB	20:39314487-39317880	-1.41324	0.00302503	-1.09403	0.00469276	-0.319213	0.999971
PRSS35	6:84222193-84235423	-0.131344	0.999971	-1.12779	0.00302503	0.996447	0.00469276
LIMCH1	4:41361623-41702061	-0.560045	0.789438	-1.1385	0.0150479	0.578454	0.77343

TNFRSF19	13:24144506-24250232	-0.357083	0.509991	-1.14053	0.00302503	0.783445	0.00469276
NHSL2	X:71130737-71381600	-1.38574	0.00302503	-1.14171	0.0416127	-0.244035	0.999971
MAN1C1	1:25943306-26112698	-1.19178	0.00302503	-1.15725	0.00302503	-0.0345257	0.999971
FGFR4	5:176513869-176525345	-0.355308	0.999971	-1.17055	0.0298268	0.815244	0.510668
TBX4	17:59529764-59562471	-1.47219	0.00302503	-1.17466	0.00302503	-0.297539	0.999971
MMP1	11:102617698-102714534	-1.86491	0.00302503	-1.18197	0.00723681	-0.682941	0.743673
LCNL1,PTGDS	9:139871955-139880862	-0.975761	0.00302503	-1.19521	0.00302503	0.21945	0.999971
COLEC12	18:319360-500722	-0.134184	0.999971	-1.21622	0.00302503	1.08204	0.00302503
ATF3	1:212738675-212794119	-0.114919	0.999971	-1.22725	0.0137916	1.11233	0.030883
A2M	12:9217772-9268825	-0.871959	0.0575555	-1.22874	0.00302503	0.356784	0.999971
SLC7A8	14:23594501-23652909	-0.588287	0.19346	-1.22971	0.00302503	0.641419	0.227773
NDNF	4:121952327-122001631	-1.01702	0.00302503	-1.24948	0.00302503	0.23246	0.999971
SCN4B	11:118004091-118023762	-0.478087	0.418586	-1.27878	0.00302503	0.800695	0.0266209
CFI	4:110661851-110723335	-0.317198	0.999971	-1.28279	0.00856372	0.965597	0.063705
ADORA1	1:203059781-203136614	-1.26392	0.00302503	-1.2921	0.00302503	0.0281808	0.999971
G0S2	1:209834708-209915895	-0.157915	0.999971	-1.319	0.00989026	1.16108	0.0244098
CELSR1	22:46755428-46933362	-0.873509	0.140139	-1.35387	0.0187202	0.480357	0.999971
SIX1	14:61110131-61125672	-0.293105	0.999971	-1.36049	0.00302503	1.06738	0.0150479
PALM	19:708952-748329	-1.59906	0.00302503	-1.36195	0.00302503	-0.237106	0.999971
MAP2	2:210288729-210599089	-0.926916	0.00856372	-1.37237	0.00302503	0.44545	0.593664
HIF3A	19:46800302-46846690	-1.2087	0.062654	-1.39047	0.030883	0.181768	0.999971
AC109642.1	2:30569354-30584134	-1.12109	0.00723681	-1.44813	0.00302503	0.327042	0.999971
APOC1	19:45417503-45422606	-0.83411	0.616315	-1.48615	0.00989026	0.652041	0.956226
DMKN	19:35988059-36004645	-0.754861	0.214483	-1.50103	0.00302503	0.74617	0.256087
XIST	X:73040285-73072588	-0.242374	0.999971	-1.52529	0.0405115	1.28291	0.112337
CD36	7:79959507-80308593	-1.20063	0.0658323	-1.52678	0.00302503	0.326154	0.999971
SEMA3F	3:49977439-50226508	-1.50488	0.0124936	-1.52978	0.0383156	0.0249019	0.999971
LRRN3	7:110302716-111202573	-0.340531	0.999971	-1.53352	0.00302503	1.19299	0.0512128
SEPP1	5:42756902-42887494	-0.74341	0.0210273	-1.5444	0.00302503	0.800991	0.00588648
CDON	11:125820010-125933230	-1.38809	0.00302503	-1.546	0.00302503	0.157909	0.999971
IGJ	4:71494460-71552533	-0.983826	0.0276966	-1.54752	0.00302503	0.563694	0.676214
UNC5C	4:96083654-96470414	-1.21554	0.00989026	-1.5544	0.00388754	0.33886	0.999971
DES	2:220283098-220291461	-1.90839	0.00302503	-1.56993	0.00302503	-0.338461	0.999971
VAMP8	2:85788684-85809154	-0.315816	0.999971	-1.59497	0.00302503	1.27916	0.0124936
TMEM37	2:120187476-120196121	-0.952097	0.38817	-1.62608	0.00588648	0.673981	0.921377
TAGLN3	3:111717510-111732734	-1.14138	0.116091	-1.68875	0.00302503	0.547373	0.999971
P2RY1	3:152552586-152559228	-0.898803	0.0150479	-1.70779	0.00302503	0.808986	0.166874
RARRES2	7:150035407-150038763	-0.884366	0.00388754	-1.71935	0.00302503	0.834982	0.0162705
C19orf33,CTB-102L5.4,SPINT2	19:38734674-38795649	-0.788092	0.898677	-1.75194	0.00469276	0.963846	0.751744

ZNF467	7:149461270-149470731	-1.15485	0.50626	-1.81364	0.00302503	0.658791	0.999971
SLC40A1	2:190425304-190448484	-0.99809	0.00302503	-1.87681	0.00302503	0.878721	0.00723681
PITX2	4:111538578-111563279	-1.69515	0.00302503	-1.87806	0.00388754	0.182918	0.999971
MCOLN2	1:85391132-85462834	-0.891509	0.184085	-1.89015	0.00302503	0.998643	0.239183
IGSF10	3:150803483-151179030	-2.19864	0.00302503	-2.04226	0.00302503	-0.156378	0.999971
COL14A1	8:121072018-121385732	-1.76617	0.00302503	-2.09567	0.00302503	0.329496	0.999971
C8orf4	8:40010988-40012821	-2.25663	0.00302503	-2.11322	0.00302503	-0.143415	0.999971
TRIM55	8:67039130-67088001	-2.60438	0.00302503	-2.16867	0.00302503	-0.435707	0.999971
SIPA1L2	1:232533558-232744697	-1.37043	0.00723681	-2.32741	0.00302503	0.956975	0.131086
GPRC5C	17:72420989-72447792	-1.61414	0.034065	-2.41437	0.00302503	0.800232	0.896417
GAP43	3:115342170-115440337	-3.33626	0.00302503	-2.87335	0.00302503	-0.462914	0.999971
MYBPH	1:203136937-203144944	-1.55666	0.132873	-3.34395	0.00302503	1.78729	0.190014
GDF10	10:48425784-48439152	-2.02977	0.00302503	-3.44738	0.00302503	1.4176	0.0491193
CHI3L1	1:203148023-203155877	-1.03411	0.00302503	-3.56539	0.00302503	2.53128	0.00302503
MYH11	16:15737123-15950890	-3.93173	0.00388754	-4.23497	0.00302503	0.303238	0.999971
IGFN1	1:201159896-201198080	-2.86057	0.00302503	-4.9559	0.00302503	2.09534	0.00302503

SSc vs Normal DEGs

		SSc vs Normal		IPF vs Normal		SSc vs IPF	
gene	locus	log2(fold_change)	q_value	log2(fold_change)	q_value	log2(fold_change)	q_value
HAPLN1	5:82933609-83017432	4.90569	0.00302503	1.52308	0.468647	3.38261	0.00302503
CXCR7	2:237476388-237491001	3.40808	0.00302503	1.13404	0.574218	2.27404	0.00302503
HHIP	4:145564073-145666423	3.27756	0.00302503	1.42024	0.099241	1.85733	0.0137916
TOX	8:59715079-60033905	2.99944	0.00302503	0.67698	0.999971	2.32246	0.00302503
RUNX3	1:25226001-25291612	2.79463	0.00302503	0.118147	0.999971	2.67648	0.00302503
WISP1	8:134203179-134243896	2.72409	0.00302503	1.24843	0.0436908	1.47566	0.00302503
CLEC14A	14:38720656-38747180	2.66734	0.00302503	2.81147	0.00302503	-0.144133	0.999971
CXCL5	4:74861358-74864496	2.56062	0.00302503	-0.517607	0.999971	3.07822	0.00302503
CHL1	3:237440-451265	2.51562	0.00302503	0.500443	0.999971	2.01518	0.00302503
PPM1H	12:63037798-63328817	2.30225	0.00302503	1.32496	0.0491193	0.977286	0.250867
AL589743.1,RP11-146E13.2,RP11-496I2.4	14:19650017-19925346	2.29558	0.00723681	2.17153	0.0137916	0.124054	0.999971
SUSD2	22:24577226-24585078	2.20665	0.00302503	-0.284084	0.999971	2.49074	0.00302503
SERPINE2	2:224839828-224904036	2.13461	0.00302503	1.87439	0.00302503	0.260226	0.999971
XG	X:2669937-2734580	2.00282	0.00469276	1.33964	0.277664	0.663184	0.990104
SFTA1P	10:10826401-10836996	1.85582	0.00388754	1.00614	0.452758	0.849684	0.565782
AP000525.8	22:16147975-16193072	1.82934	0.00302503	1.82142	0.00302503	0.0079158	0.999971
AMIGO2	12:47469489-47630443	1.82472	0.00302503	1.2227	0.00302503	0.602022	0.2818
KISS1	1:204159468-204165614	1.76444	0.0276966	2.93945	0.00302503	-1.17501	0.0544352
FMO3	1:171060010-171087008	1.7533	0.00302503	1.64992	0.00302503	0.103374	0.999971

GLIS1	1:53971875-54204819	1.65244	0.0137916	1.31816	0.0790884	0.334281	0.999971
SLC7A11	4:138948575-139163580	1.56363	0.00302503	0.576032	0.351083	0.987601	0.0112285
WFDC1	16:84328229-84363457	1.51177	0.00302503	1.42874	0.00302503	0.0830339	0.999971
ALDH1A1	9:75515577-75695358	1.51145	0.00302503	0.187107	0.999971	1.32435	0.00302503
PLCB4	20:9049351-9461889	1.50913	0.00302503	1.64647	0.00302503	-0.13734	0.999971
SULF1	8:70378858-70573150	1.49896	0.00302503	1.08998	0.00388754	0.408984	0.896417
NEK10	3:27148387-27411067	1.48001	0.00302503	0.949507	0.0616427	0.530499	0.628053
SLC16A14	2:230899486-230934095	1.46758	0.00388754	0.00610671	0.999971	1.46148	0.00469276
ADRA1D	20:4201236-4230191	1.45214	0.0112285	2.2127	0.00302503	-0.760564	0.382056
PSG1,PSG7,PSG8	19:43256837-43441330	1.4418	0.00302503	1.14606	0.0150479	0.295732	0.999971
PSG9	19:43715651-43773682	1.44172	0.00302503	0.50763	0.875972	0.934093	0.0330131
ANO4	12:101111149-101522419	1.43445	0.00302503	0.347592	0.999971	1.08686	0.141037
PTGS1	9:125132823-125158266	1.42547	0.00302503	0.723927	0.00469276	0.701545	0.00388754
CTD-2319I12.1	17:58156669-58169246	1.38779	0.00588648	-0.172737	0.999971	1.56053	0.00388754
KCNJ2	17:68163101-68176189	1.38508	0.00302503	0.679706	0.448139	0.705372	0.226299
TSPAN13	7:16793159-16824203	1.37264	0.00302503	0.738973	0.0351647	0.633664	0.0669097
AP000688.8	21:37376893-37394325	1.32709	0.00302503	0.743097	0.322122	0.583995	0.425578
OSR2	8:99956630-99964503	1.31697	0.00302503	0.325218	0.999971	0.991753	0.0850954
ANGPT1	8:108246611-108510300	1.3149	0.00302503	0.403692	0.54333	0.911212	0.00302503
GPR37	7:124384367-124406040	1.30867	0.00302503	1.06721	0.00989026	0.241457	0.999971
CHRM2	7:136370305-136866854	1.22619	0.00989026	0.734531	0.297695	0.491664	0.727134
SNAP25	20:9966735-10349424	1.21191	0.0124936	0.922783	0.105993	0.289123	0.999971
IGFBP2	2:217497550-217529159	1.17919	0.00588648	0.85445	0.109563	0.32474	0.999971
WNT5A	3:55499740-55523973	1.17421	0.00302503	1.24029	0.00302503	-0.0660868	0.999971
EPHB2	1:23037331-23241818	1.17205	0.00302503	0.271816	0.896579	0.900232	0.00302503
SEMA6D	15:47476297-48066420	1.13362	0.0351647	0.527653	0.801914	0.605964	0.503241
BMP4	14:54416453-54425479	1.11858	0.0112285	0.425643	0.999971	0.692935	0.262027
LRRN4CL	11:62453873-62457371	1.08596	0.00302503	0.730396	0.0964313	0.355559	0.896417
PLOD2	3:145777312-145881440	1.08195	0.00302503	0.966321	0.00302503	0.115627	0.999971
SRGN	10:70847861-70864567	1.06947	0.00302503	0.710457	0.00302503	0.359012	0.304714
CORIN	4:47595959-47840385	1.063	0.034065	1.88389	0.00302503	-0.820891	0.0276966
CPNE7	16:89642175-89663654	1.04916	0.00302503	1.08782	0.00302503	-0.0386525	0.999971
COL8A1	3:99357298-99518070	1.04466	0.00302503	1.04585	0.00302503	-0.00119162	0.999971
FMN2	1:240177647-240643504	1.03789	0.0112285	0.793293	0.168499	0.244593	0.999971
FAM69A	1:93307723-93427057	1.03643	0.00302503	0.499858	0.224592	0.53657	0.0709936
GPNMB	7:23275585-23314727	1.03451	0.00302503	0.544574	0.0198838	0.489935	0.0394198
CHAC1	15:41245159-41248710	0.977631	0.00302503	0.631254	0.140139	0.346377	0.879342
TLE4	9:82186647-82341779	0.963763	0.0112285	0.771467	0.0770038	0.192296	0.999971
LIMS2	2:128395955-128439360	0.952511	0.0124936	1.13451	0.00302503	-0.182004	0.999971

DHRS3	1:12627938-12679612	0.925434	0.00302503	0.0311934	0.999971	0.89424	0.00388754
MASP1	3:186935941-187009810	0.91987	0.0112285	0.414616	0.896417	0.505255	0.540968
AMPH	7:38423304-38671167	0.918888	0.00302503	0.834399	0.0175003	0.0844891	0.999971
RND3	2:151324708-151395525	0.917223	0.00302503	0.584009	0.00588648	0.333214	0.517505
TGFBI	5:135364583-135399507	0.910227	0.00302503	0.348519	0.582247	0.561708	0.126443
SLC9A3R2	16:2075356-2089027	0.901016	0.00302503	0.407535	0.250867	0.49348	0.0501908
EGFL7	9:139543061-139567131	0.899864	0.0426246	0.577265	0.566761	0.322599	0.999971
LEPREL1	3:189674510-189862635	0.896189	0.00302503	0.722134	0.00989026	0.174055	0.999971
NEDD9	6:11173684-11382581	0.895117	0.00302503	0.936184	0.00302503	-0.0410672	0.999971
FAM167A	8:11197145-11333407	0.887282	0.00388754	0.606888	0.176344	0.280394	0.999971
APBB1IP	10:26727090-26857339	0.876469	0.0383156	0.231976	0.999971	0.644493	0.258237
SERPINF1	17:1665252-1681183	0.871195	0.00302503	-0.391376	0.445056	1.26257	0.00302503
LOX	5:121297655-121414246	0.870875	0.00302503	1.31352	0.00302503	-0.442642	0.642502
RGCC	13:42031665-42045018	0.864313	0.0137916	0.388769	0.939229	0.475544	0.622834
KANK1	9:213107-746105	0.862027	0.00302503	0.573613	0.0383156	0.288414	0.855811
FAM101B	17:289768-295730	0.851016	0.00302503	0.802211	0.00302503	0.0488044	0.999971
SLC2A1	1:43391518-43449029	0.847984	0.00302503	0.699794	0.00302503	0.14819	0.999971
SLC16A3	17:80186292-80231607	0.83945	0.00302503	0.861532	0.00302503	-0.0220827	0.999971
CHN1	2:175664090-175870106	0.837281	0.00302503	0.79232	0.00302503	0.0449605	0.999971
STC2	5:172741659-172756506	0.823539	0.0330131	0.437086	0.765941	0.386453	0.934394
S100A4	1:153516088-153524887	0.813798	0.00302503	-0.182726	0.999971	0.996523	0.00302503
BICC1	10:60272754-60591195	0.809875	0.00302503	0.341444	0.55573	0.468431	0.107802
MYO1D	17:30819539-31205655	0.80743	0.0244098	0.471513	0.59774	0.335917	0.896417
MID1	X:10413349-10851773	0.793894	0.00302503	0.223989	0.999971	0.569905	0.00989026
CDH6	5:31094083-31329253	0.793604	0.00302503	-0.360069	0.729694	1.15367	0.00302503
RCN3	19:50030874-50050219	0.77507	0.00302503	0.483236	0.0351647	0.291834	0.560632
TMEM45A	3:100211025-100296289	0.747777	0.00588648	0.66891	0.0221778	0.0788676	0.999971
PALLD	4:169246986-169931426	0.729434	0.00302503	0.48341	0.0501908	0.246024	0.896417
DCN	12:91539024-91576900	0.727484	0.00388754	0.317122	0.820957	0.410362	0.50047
PVRL3	3:110607230-110994410	0.720104	0.00723681	0.619933	0.0436908	0.100172	0.999971
DUSP10	1:221874765-221915518	0.68783	0.00469276	0.797301	0.00302503	-0.109471	0.999971
SERINC2	1:31882411-31907525	0.663769	0.00302503	1.02403	0.00302503	-0.360261	0.347042
F3	1:94993738-95008728	0.654209	0.00388754	0.552419	0.0319506	0.10179	0.999971
ID1	20:30181441-30194318	0.641282	0.00302503	0.409179	0.184085	0.232103	0.896417
CLDN11	3:170136652-170578169	-0.605413	0.00302503	-0.00824016	0.999971	-0.597172	0.00302503
GEM	8:95261478-95274578	-0.693816	0.00388754	-0.259613	0.894717	-0.434203	0.204096
HLA-A	6:29855070-29913661	-0.703213	0.00302503	-0.314701	0.767687	-0.388512	0.392301
CORO2B	15:68871307-69020145	-0.713295	0.00302503	-0.493489	0.0288343	-0.219806	0.95797
SDPR	2:192699027-193060490	-0.71856	0.00723681	-0.0747569	0.999971	-0.643803	0.0137916

KRT19	17:39679868-39684693	-0.721253	0.00856372	-0.307106	0.896417	-0.414147	0.669306
MDGA1	6:37598454-37667082	-0.735376	0.00388754	-0.627497	0.0124936	-0.107878	0.999971
MARCKSL1	1:32799432-32801980	-0.740621	0.00302503	-0.52477	0.0276966	-0.215851	0.999971
SLIT2	4:20253351-20623803	-0.741785	0.00723681	-0.6899	0.0150479	-0.0518853	0.999971
EFEMP1	2:56093101-56151274	-0.743544	0.00302503	-0.64853	0.00302503	-0.095014	0.999971
SCARA3	8:27491384-27534190	-0.753844	0.00302503	-0.117969	0.999971	-0.635875	0.00989026
GPR126	6:142622990-142767652	-0.75515	0.0480437	-0.4028	0.645311	-0.35235	0.950059
GDF5	20:34020826-34042568	-0.783599	0.00989026	-0.507057	0.247107	-0.276542	0.999971
ENPP2	8:120569323-120685693	-0.793009	0.00302503	-0.992212	0.00302503	0.199204	0.999971
SFRP1	8:41119480-41167016	-0.795026	0.00302503	-0.694752	0.00723681	-0.100273	0.999971
RRAD	16:66955581-66959547	-0.80254	0.0298268	-0.0989519	0.999971	-0.703588	0.168499
PBX1	1:164524820-164868533	-0.837945	0.00469276	-0.999217	0.00302503	0.161272	0.999971
CDCA7	2:174202946-174233725	-0.841555	0.0298268	-0.820206	0.0298268	-0.0213485	0.999971
ABLIM1	10:116190808-116539662	-0.883883	0.0426246	-0.781176	0.0575555	-0.102707	0.999971
RARRES2	7:150035407-150038763	-0.884366	0.00388754	-1.71935	0.00302503	0.834982	0.0162705
FNBP1L	1:93913586-94020356	-0.88512	0.00302503	-0.512738	0.258237	-0.372382	0.847124
CSRP2	12:77252002-77274132	-0.89494	0.00302503	-0.835992	0.00302503	-0.0589481	0.999971
P2RY1	3:152552586-152559228	-0.898803	0.0150479	-1.70779	0.00302503	0.808986	0.166874
OSBPL8	12:76745310-76953589	-0.913699	0.00856372	-0.748986	0.0276966	-0.164713	0.999971
MAP2	2:210288729-210599089	-0.926916	0.00856372	-1.37237	0.00302503	0.44545	0.593664
NFIB	9:14081841-14398982	-0.949975	0.00302503	-0.947899	0.00302503	-0.00207633	0.999971
LCNL1,PTGDS	9:139871955-139880862	-0.975761	0.00302503	-1.19521	0.00302503	0.21945	0.999971
IGJ	4:71494460-71552533	-0.983826	0.0276966	-1.54752	0.00302503	0.563694	0.676214
F2RL1	5:76114680-76131214	-0.996228	0.00302503	-0.675349	0.025545	-0.320878	0.961697
SLC40A1	2:190425304-190448484	-0.99809	0.00302503	-1.87681	0.00302503	0.878721	0.00723681
NDNF	4:121952327-122001631	-1.01702	0.00302503	-1.24948	0.00302503	0.23246	0.999971
RNF128	X:105937023-106040223	-1.03593	0.00302503	-0.19563	0.999971	-0.840298	0.00723681
SNED1	2:241938157-242041747	-1.04794	0.0351647	-0.842998	0.2956	-0.204938	0.999971
ADAMTS15	11:130317906-130346541	-1.07791	0.00302503	-1.06821	0.00302503	-0.00969856	0.999971
RHOJ	14:63670831-63760353	-1.07992	0.00388754	-0.594038	0.364149	-0.485879	0.825553
CLU	8:27454433-27472548	-1.07994	0.00302503	0.106592	0.999971	-1.18654	0.00388754
AC109642.1	2:30569354-30584134	-1.12109	0.00723681	-1.44813	0.00302503	0.327042	0.999971
ESM1	5:54273691-54330398	-1.12727	0.00302503	-0.0270261	0.999971	-1.10024	0.00302503
ADCY8	8:131792546-132054764	-1.12903	0.0175003	-0.958707	0.0426246	-0.170322	0.999971
PODXL	7:131184632-131242976	-1.12978	0.00302503	-0.363711	0.790621	-0.766071	0.059618
DCHS1	11:6642555-6677573	-1.13556	0.00302503	-0.905467	0.00302503	-0.23009	0.999971
MAOB	X:43625772-43741693	-1.13845	0.00302503	-0.87574	0.00469276	-0.262706	0.999971
MAN1C1	1:25943306-26112698	-1.19178	0.00302503	-1.15725	0.00302503	-0.0345257	0.999971
TMSB15A	X:101768603-101771712	-1.19691	0.00588648	-0.568347	0.612937	-0.628558	0.615367

TNC	9:117782805-117880765	-1.21006	0.00302503	-0.861244	0.00388754	-0.348821	0.844404
UNC5C	4:96083654-96470414	-1.21554	0.00989026	-1.5544	0.00388754	0.33886	0.999971
SEMA3D	7:84624352-84816355	-1.25089	0.0198838	-0.263978	0.999971	-0.986914	0.152107
ADORA1	1:203059781-203136614	-1.26392	0.00302503	-1.2921	0.00302503	0.0281808	0.999971
APOE	19:45409010-45412650	-1.27245	0.00302503	-0.296156	0.999971	-0.976297	0.0362438
NHSL2	X:71130737-71381600	-1.38574	0.00302503	-1.14171	0.0416127	-0.244035	0.999971
CDON	11:125820010-125933230	-1.38809	0.00302503	-1.546	0.00302503	0.157909	0.999971
MAFB	20:39314487-39317880	-1.41324	0.00302503	-1.09403	0.00469276	-0.319213	0.999971
CADM1	11:115039937-115375675	-1.43442	0.00302503	-0.313234	0.999971	-1.12118	0.00588648
TBX4	17:59529764-59562471	-1.47219	0.00302503	-1.17466	0.00302503	-0.297539	0.999971
NCAM1	11:112830001-113185159	-1.47331	0.00388754	-0.187486	0.999971	-1.28583	0.0288343
FAM213A	10:82167584-82195320	-1.47801	0.00302503	-0.959602	0.030883	-0.518405	0.852231
VSNL1	2:17719976-17838285	-1.48555	0.00856372	-0.288147	0.999971	-1.19741	0.098291
SEMA3F	3:49977439-50226508	-1.50488	0.0124936	-1.52978	0.0383156	0.0249019	0.999971
NR4A3	9:102583950-102629249	-1.50936	0.00302503	-0.556109	0.57501	-0.953248	0.0740196
NCAM2	21:22370604-22915650	-1.5389	0.00302503	-0.317883	0.999971	-1.22101	0.00856372
PALM	19:708952-748329	-1.59906	0.00302503	-1.36195	0.00302503	-0.237106	0.999971
GPRC5C	17:72420989-72447792	-1.61414	0.034065	-2.41437	0.00302503	0.800232	0.896417
PITX2	4:111538578-111563279	-1.69515	0.00302503	-1.87806	0.00388754	0.182918	0.999971
COL14A1	8:121072018-121385732	-1.76617	0.00302503	-2.09567	0.00302503	0.329496	0.999971
FGF18	5:170845578-170884627	-1.83263	0.00588648	-0.891826	0.465343	-0.940801	0.538089
MMP1	11:102617698-102714534	-1.86491	0.00302503	-1.18197	0.00723681	-0.682941	0.743673
DES	2:220283098-220291461	-1.90839	0.00302503	-1.56993	0.00302503	-0.338461	0.999971
NDP	X:43808021-43832939	-2.00174	0.00302503	-0.651735	0.462013	-1.35001	0.0124936
GDF10	10:48425784-48439152	-2.02977	0.00302503	-3.44738	0.00302503	1.4176	0.0491193
IGSF10	3:150803483-151179030	-2.19864	0.00302503	-2.04226	0.00302503	-0.156378	0.999971
RPL9	4:39453853-39460568	-2.20071	0.00302503	-0.333382	0.999971	-1.86733	0.00302503
C8orf4	8:40010988-40012821	-2.25663	0.00302503	-2.11322	0.00302503	-0.143415	0.999971
LAMP5	20:9485826-9511171	-2.54364	0.00302503	-1.09282	0.00302503	-1.45083	0.00302503
TRIM55	8:67039130-67088001	-2.60438	0.00302503	-2.16867	0.00302503	-0.435707	0.999971
CHRDL1	X:109916844-110039504	-2.67569	0.00302503	-0.814892	0.169321	-1.8608	0.00302503
IGFN1	1:201159896-201198080	-2.86057	0.00302503	-4.9559	0.00302503	2.09534	0.00302503
FXYD2,FXYD6,FXYD6-FXYD2	11:117298488-117748201	-2.93575	0.00302503	-1.00111	0.785179	-1.93464	0.0800398
RORB	9:77100466-77308094	-3.04905	0.00302503	-0.972425	0.223899	-2.07663	0.12833
GAP43	3:115342170-115440337	-3.33626	0.00302503	-2.87335	0.00302503	-0.462914	0.999971
MYH11	16:15737123-15950890	-3.93173	0.00388754	-4.23497	0.00302503	0.303238	0.999971
SLCO2A1	3:133651539-133771028	-4.02305	0.00302503	-1.14208	0.299661	-2.88098	0.00302503

SSc vs IPF DEGs

		SSc vs Normal		IPF vs Normal		SSc vs IPF	
gene	locus	log2(fold_change)	q_value	log2(fold_change)	q_value	log2(fold_change)	q_value
IL8	4:74606222-74609433	2.6777	0.00302503	-0.917577	0.55653	3.59528	0.00302503
CXCL5	4:74861358-74864496	2.56062	0.00302503	-0.517607	0.999971	3.07822	0.00302503
TMEM176A	7:150488372-150502208	0.731282	0.999971	-2.13973	0.0124936	2.87101	0.0137916
EDNRB	13:78469615-78493903	1.17874	0.0288343	-1.51349	0.0501908	2.69223	0.00302503
RUNX3	1:25226001-25291612	2.79463	0.00302503	0.118147	0.999971	2.67648	0.00302503
CHI3L1	1:203148023-203155877	-1.03411	0.00302503	-3.56539	0.00302503	2.53128	0.00302503
SUSD2	22:24577226-24585078	2.20665	0.00302503	-0.284084	0.999971	2.49074	0.00302503
TOX	8:59715079-60033905	2.99944	0.00302503	0.67698	0.999971	2.32246	0.00302503
CHL1	3:237440-451265	2.51562	0.00302503	0.500443	0.999971	2.01518	0.00302503
LIMS3	2:110656004-110742667	0.736754	0.606995	-0.865708	0.678526	1.60246	0.0319506
CTD-2319I12.1	17:58156669-58169246	1.38779	0.00588648	-0.172737	0.999971	1.56053	0.00388754
INSC	11:15133969-15291142	1.67843	0.0909025	0.127049	0.999971	1.55138	0.0233069
SLC16A14	2:230899486-230934095	1.46758	0.00388754	0.00610671	0.999971	1.46148	0.00469276
GDF10	10:48425784-48439152	-2.02977	0.00302503	-3.44738	0.00302503	1.4176	0.0491193
TMEM119	12:108983621-108992096	0.50707	0.0890229	-0.824454	0.00302503	1.33152	0.00302503
ALDH1A1	9:75515577-75695358	1.51145	0.00302503	0.187107	0.999971	1.32435	0.00302503
VAMP8	2:85788684-85809154	-0.315816	0.999971	-1.59497	0.00302503	1.27916	0.0124936
SERPINF1	17:1665252-1681183	0.871195	0.00302503	-0.391376	0.445056	1.26257	0.00302503
BDKRB1,BDKRB2,RP11-404P21.8	14:96671015-96735304	0.663952	0.135538	-0.566953	0.29089	1.2309	0.00302503
PEAR1	1:156863148-156886226	0.532751	0.144725	-0.697383	0.0319506	1.23013	0.00302503
G0S2	1:209834708-209915895	-0.157915	0.999971	-1.319	0.00989026	1.16108	0.0244098
CDH6	5:31094083-31329253	0.793604	0.00302503	-0.360069	0.729694	1.15367	0.00302503
AKR1C3	10:5077548-5149878	0.493203	0.39349	-0.63387	0.237713	1.12707	0.00302503
COLEC12	18:319360-500722	-0.134184	0.999971	-1.21622	0.00302503	1.08204	0.00302503
LDB2	4:16402052-16900432	0.631239	0.348742	-0.436984	0.994636	1.06822	0.0351647
SIX1	14:61110131-61125672	-0.293105	0.999971	-1.36049	0.00302503	1.06738	0.0150479
VWA5A	11:123986068-124018428	0.424423	0.638517	-0.602565	0.19265	1.02699	0.00302503
S100A4	1:153516088-153524887	0.813798	0.00302503	-0.182726	0.999971	0.996523	0.00302503
PIR	X:15402920-15574652	0.8832	0.0298268	-0.0958849	0.999971	0.979085	0.0175003
PCDH18	4:138440071-138453804	0.658269	0.00588648	-0.31637	0.7145	0.974639	0.00302503
PRKG2	4:82007662-82137627	0.0254716	0.999971	-0.940984	0.00302503	0.966456	0.0162705
TNFRSF21	6:47199267-47277735	0.228251	0.999971	-0.70709	0.0298268	0.935342	0.00302503
ANGPT1	8:108246611-108510300	1.3149	0.00302503	0.403692	0.54333	0.911212	0.00302503
EPHB2	1:23037331-23241818	1.17205	0.00302503	0.271816	0.896579	0.900232	0.00302503
DHRS3	1:12627938-12679612	0.925434	0.00302503	0.0311934	0.999971	0.89424	0.00388754
FBLN1	22:45898117-45997044	0.467574	0.190941	-0.413248	0.322122	0.880822	0.00302503
SLC40A1	2:190425304-190448484	-0.99809	0.00302503	-1.87681	0.00302503	0.878721	0.00723681

NQO1	16:69740898-69760854	0.774441	0.00302503	-0.103614	0.999971	0.878055	0.00302503
GUCY1B3	4:156680032-156728746	0.0368626	0.999971	-0.831847	0.0426246	0.86871	0.0372946
SCN4B	11:118004091-118023762	-0.478087	0.418586	-1.27878	0.00302503	0.800695	0.0266209
TNFRSF19	13:24144506-24250232	-0.357083	0.509991	-1.14053	0.00302503	0.783445	0.00469276
AKR1C1	10:4934795-5060207	0.200264	0.999971	-0.541462	0.162493	0.741726	0.00856372
PTGS1	9:125132823-125158266	1.42547	0.00302503	0.723927	0.00469276	0.701545	0.00388754
HMOX1	22:35776353-35790207	0.578305	0.00469276	-0.116513	0.999971	0.694818	0.00302503
CLDN11	3:170136652-170578169	-0.605413	0.00302503	-0.00824016	0.999971	-0.597172	0.00302503
SDPR	2:192699027-193060490	-0.71856	0.00723681	-0.0747569	0.999971	-0.643803	0.0137916
RPS4Y1	Y:2709526-2800041	0.0326085	0.999971	0.702878	0.0586144	-0.670269	0.0458797
MAMDC2	9:72658496-72873782	0.0557389	0.999971	0.813716	0.00723681	-0.757977	0.0266209
IGFBP5	2:217536827-217858722	0.236752	0.999971	1.05428	0.00302503	-0.81753	0.00302503
RNF128	X:105937023-106040223	-1.03593	0.00302503	-0.19563	0.999971	-0.840298	0.00723681
CDH13	16:82660407-83841439	-0.37212	0.896417	0.554625	0.114199	-0.926745	0.0162705
HOPX	4:57514154-57548065	-0.743457	0.245735	0.217247	0.999971	-0.960704	0.0447753
CTSH	15:79213399-79241916	-0.15947	0.999971	0.873137	0.132873	-1.03261	0.0362438
SSTR1	14:38675857-38682272	0.0621314	0.999971	1.14106	0.00302503	-1.07893	0.00302503
ESM1	5:54273691-54330398	-1.12727	0.00302503	-0.0270261	0.999971	-1.10024	0.00302503
CADM1	11:115039937-115375675	-1.43442	0.00302503	-0.313234	0.999971	-1.12118	0.00588648
SYNPO2	4:119809995-119982402	-0.386278	0.672307	0.804673	0.00302503	-1.19095	0.00302503
OXTR	3:8661085-9005457	-0.0877144	0.999971	1.17228	0.0890229	-1.25999	0.0288343
BEX1	X:102317578-102319168	0.317879	0.999971	1.70896	0.00723681	-1.39108	0.00856372
NRXN3	14:78708733-80331052	-1.14913	0.00856372	0.251236	0.999971	-1.40036	0.00302503
TPTEP1	22:17082231-17185367	-0.961054	0.258237	0.682343	0.250867	-1.6434	0.00302503
RPL9	4:39453853-39460568	-2.20071	0.00302503	-0.333382	0.999971	-1.86733	0.00302503
DSG2	18:29078005-29136874	-0.69424	0.68105	1.18478	0.00302503	-1.87902	0.00469276
GRPR	X:16026785-16189587	-1.40371	0.0955995	0.489856	0.999971	-1.89357	0.00469276
KPRP	1:152730505-152734529	-1.43901	0.0298268	0.571522	0.999971	-2.01053	0.00388754
GPAT2	2:96676307-96705199	-0.176993	0.999971	1.89291	0.0137916	-2.0699	0.0150479

Table S5

Differentially expressed miRNAs across all patient lung fibroblast comparisons (≥ + 1.35-fold or ≤ -1.35-fold, *p* < 0.1)

miRNA ID<br><br>*IPF vs Ctl (significant)*	Fold change	*p*-value	Association with fibrosis
hsa-miR-138-5p	-1.96	8.71E-02	Inhibits human hypertrophic scar fibroblast proliferation and movement in vitro[8]. Inhibits the osteogenic differentiation of hMSCs and bone formation in vivo[9,10]. Suppresses epithelial-mesenchymal transition in squamous cell carcinoma cell lines[11]. Decreased expression in IPF lung[12].
hsa-miR-146b-5p	-1.86	4.66E-03	Targets SMAD4 and affects TGF-β signaling[13]. Expression reduced in CAFs and inhibits activation, migration, and invasion of breast stromal fibroblasts[14]. Closely related miR-146a has been shown to inhibit TGF-β-mediated activation of dermal fibroblasts and HSCs, and inhibited renal fibrosis in UUO model[15–17].
hsa-miR-155-5p	-1.83	1.38E-03	Downregulated by TGF-β in lung fibroblasts[18]. Targets SMAD2 and affects TGF-β-dependent gene expression in THP1 cells, however was found to facilitate TGF-β-induced EMT in mammary gland epithelial cells[19,20]. Increased expression in IPF lung[12].
hsa-miR-190a-5p	-1.69	4.38E-04	Inhibits TGF-β signaling[21].
hsa-miR-125b-2-3p	-1.62	1.83E-03	
hsa-miR-708-3p	-1.59	4.07E-03	

hsa-miR-20a-5p	-1.53	3.60E-03	Part of mir17~92 cluster. Cluster is downregulated in IPF lung and fibroblasts. mir17~92 cluster reverses IPF fibroblast activation[22].
hsa-miR-374a-3p	-1.52	2.04E-03	
hsa-miR-21-3p	-1.49	2.18E-02	
hsa-miR-340-5p	-1.46	4.18E-03	Upregulated in liver fibrosis models[23]. Enhances TGF-β signaling[21].
hsa-miR-17-5p	-1.43	1.58E-03	Part of mir17~92 cluster. Cluster is downregulated in IPF lung and fibroblasts. mir17~92 cluster reverses IPF fibroblast activation[22].
hsa-miR-1307-5p	-1.43	2.17E-02	
hsa-miR-99a-5p	-1.42	9.70E-03	
hsa-miR-345-5p	-1.40	1.52E-02	Targets SMAD1[24].
hsa-miR-7974	-1.39	7.45E-02	
hsa-miR-93-5p	-1.37	1.16E-02	Modulates TGF-β signaling[21,25–28]. Targets SMAD7, TGFβR2[26,28]. Downregulated in slowly progressive IPF lung[29].
hsa-miR-335-5p	2.27	9.45E-02	Levels reduced during HSC activation, inhibits HSC migration/activation[30]. Plays a role in osteogenic differentiation and chondrogenesis[31,32].
hsa-miR-30a-3p	1.38	5.48E-02	Reduced expression in IPF lung[33].
hsa-miR-574-3p	1.37	7.89E-03	
SSc vs Ctl (significant)			
hsa-miR-146b-5p	-1.93	3.01E-03	Targets SMAD4 and affects TGF-β signaling[13]. Expression reduced in CAFs and inhibits activation, migration, and invasion of breast stromal fibroblasts[14]. Closely related miR-146a has been shown to inhibit TGF-β-mediated activation of dermal fibroblasts and HSCs, and inhibited renal fibrosis in UUO model[15–17].
hsa-miR-708-3p	-1.59	4.10E-03	

hsa-miR-20a-5p	-1.50	5.47E-03	Part of mir17~92 cluster. Cluster is downregulated in IPF lung and fibroblasts. mir17~92 cluster reverses IPF fibroblast activation[22].
hsa-miR-21-3p	-1.47	2.46E-02	
hsa-miR-17-5p	-1.44	1.32E-03	Part of mir17~92 cluster. Cluster is downregulated in IPF lung and fibroblasts. mir17~92 cluster reverses IPF fibroblast activation[22].
hsa-miR-190a-5p	-1.44	1.02E-02	Inhibits TGF-β signaling[21].
hsa-miR-155-5p	-1.43	4.74E-02	Downregulated by TGF-β in lung fibroblasts[18]. Targets SMAD2 and affects TGF-β-dependent gene expression in THP1 cells, however was found to facilitate TGF-β-induced EMT in mammary gland epithelial cells[19,20]. Increased expression in IPF lung[12].
hsa-miR-29b-3p	-1.38	7.31E-02	miR-29 family is downregulated in several fibrosis models and human fibrotic disease. Attenuates fibrosis in mouse models of lung, cardiac, liver, and kidney fibrosis. Affects fibroblast/HSC activity. Targets ECM and pro-fibrotic pathway genes[12,34–46].
hsa-miR-345-5p	-1.38	1.96E-02	Targets SMAD1[24].
hsa-miR-101-3p	-1.38	6.53E-03	Inhibits TGF-β-induced EMT in hepatocytes[47]. Attenuates TGF-β pathway and targets TGFβR1. Suppresses liver fibrosis in mice and promotes HSC deactivation in vitro[48]. Downregulated in serum and lung of IPF patients[29,49]. Inhibited post infarct cardiac fibrosis in rat MI model[50].
hsa-miR-374a-3p	-1.38	1.49E-02	
hsa-miR-340-5p	-1.35	1.86E-02	Upregulated in liver fibrosis models[23]. Enhances TGF-β signaling[21].

hsa-miR-199b-5p	1.80	1.76E-02	Increased expression of miR-199a in CCL4-induced liver fibrosis and human liver fibrosis. miR-199a increases expression of fibrosis genes in HSCs[51]. miR-199a mediates TGF-β-induced activation of lung fibroblasts[52]. Increased expression in IPF lung[33].
hsa-miR-486-5p	1.72	4.53E-02	Downregulated in mouse models of lung fibrosis and in IPF. Mimics inhibited a mouse model of lung fibrosis and TGF-β-induced fibrogenesis in mouse fibroblasts. Targets SMAD2[53].
hsa-miR-431-5p	1.61	1.16E-03	
hsa-miR-432-5p	1.50	1.16E-03	Increased expression in IPF lungs[33].
hsa-miR-654-3p	1.49	1.90E-04	Increased expression in IPF lungs[33].
hsa-miR-127-3p	1.45	1.32E-03	Increased expression in IPF lungs[33].
hsa-miR-409-3p	1.45	1.91E-03	Increased expression in IPF lungs[33].
hsa-miR-370-3p	1.42	3.92E-04	Reduced expression in CCL_4 liver fibrosis and TGF-β-stimulated HSCs. Suppressed activation of HSCs and attenuated CCL_4-induced liver fibrosis[54]. Increased expression in IPF lungs[33].
hsa-miR-134-5p	1.41	7.27E-04	Increased expression in IPF lungs[12].
hsa-miR-493-3p	1.40	8.74E-04	
hsa-miR-410-3p	1.36	4.10E-03	Increased expression in IPF lungs[33].
hsa-miR-411-5p	1.35	7.37E-03	Increased expression in IPF lungs[33].
IPF vs SSc (significant)			
hsa-miR-125b-2-3p	-1.65	1.23E-03	
hsa-miR-99a-5p	-1.59	9.44E-04	
hsa-miR-199b-5p	-1.57	6.52E-02	Increased expression of miR-199a in CCL4-induced liver fibrosis and human liver fibrosis. miR-199a increases expression of fibrosis genes in HSCs[51]. miR-199a mediates TGF-β-induced activation of lung fibroblasts[52]. Increased expression in IPF lung[33].
hsa-miR-431-5p	-1.42	1.36E-02	

hsa-miR-381-3p	-1.39	1.08E-02	Increased expression in IPF lungs[33].
hsa-miR-411-5p	-1.37	6.02E-03	Increased expression in IPF lungs[33].
hsa-miR-335-5p	2.24	9.85E-02	Levels reduced during HSC activation, inhibits HSC migration/activation[30]. Plays a role in osteogenic differentiation and chondrogenesis[31,32].

1. Hsu, E.; Shi, H.; Jordan, R. M.; Lyons-Weiler, J.; Pilewski, J. M.; Feghali-Bostwick, C. A. Lung tissues in patients with systemic sclerosis have gene expression patterns unique to pulmonary fibrosis and pulmonary hypertension. *Arthritis Rheum* **2011**, *63*, 783–94, doi:10.1002/art.30159.
2. Huang da, W.; Sherman, B. T.; Lempicki, R. A. Systematic and integrative analysis of large gene lists using DAVID bioinformatics resources. *Nat Protoc* **2009**, *4*, 44–57, doi:10.1038/nprot.2008.211.
3. Supek, F.; Bosnjak, M.; Skunca, N.; Smuc, T. REVIGO summarizes and visualizes long lists of gene ontology terms. *PLoS One* **2011**, *6*, e21800, doi:10.1371/journal.pone.0021800.
4. Wettlaufer, S. H.; Scott, J. P.; McEachin, R. C.; Peters-Golden, M.; Huang, S. K. Myofibroblast Dedifferentiation by PGE is Characterized by Reversal of the Global Transcriptome. *Am J Respir Cell Mol Biol* **2015**, doi:10.1165/rcmb.2014-0468OC.
5. Subramanian, A.; Tamayo, P.; Mootha, V. K.; Mukherjee, S.; Ebert, B. L.; Gillette, M. A.; Paulovich, A.; Pomeroy, S. L.; Golub, T. R.; Lander, E. S.; Mesirov, J. P. Gene set enrichment analysis: a knowledge-based approach for interpreting genome-wide expression profiles. *Proc Natl Acad Sci U A* **2005**, *102*, 15545–50, doi:10.1073/pnas.0506580102.
6. Didangelos, A.; Yin, X.; Mandal, K.; Baumert, M.; Jahangiri, M.; Mayr, M. Proteomics characterization of extracellular space components in the human aorta. *Mol Cell Proteomics* **2010**, *9*, 2048–62, doi:10.1074/mcp.M110.001693.
7. Decaris, M. L.; Gatmaitan, M.; FlorCruz, S.; Luo, F.; Li, K.; Holmes, W. E.; Hellerstein, M. K.; Turner, S. M.; Emson, C. L. Proteomic analysis of altered extracellular matrix turnover in bleomycin-induced pulmonary fibrosis. *Mol Cell Proteomics* **2014**, *13*, 1741–52, doi:10.1074/mcp.M113.037267.
8. Xiao, Y. Y.; Fan, P. J.; Lei, S. R.; Qi, M.; Yang, X. H. MiR-138/peroxisome proliferator-activated receptor beta signaling regulates human hypertrophic scar fibroblast proliferation and movement in vitro. *J Dermatol* **2015**, *42*, 485–95, doi:10.1111/1346-8138.12792.
9. Eskildsen, T.; Taipaleenmaki, H.; Stenvang, J.; Abdallah, B. M.; Ditzel, N.; Nossent, A. Y.; Bak, M.; Kauppinen, S.; Kassem, M. MicroRNA-138 regulates osteogenic differentiation of human stromal (mesenchymal) stem cells in vivo. *Proc Natl Acad Sci U A* **2011**, *108*, 6139–44, doi:10.1073/pnas.1016758108.
10. Qu, B.; Xia, X.; Wu, H. H.; Tu, C. Q.; Pan, X. M. PDGF-regulated miRNA-138 inhibits the osteogenic differentiation of mesenchymal stem cells. *Biochem Biophys Res Commun* **2014**, *448*, 241–7, doi:10.1016/j.bbrc.2014.04.091.
11. Liu, X.; Wang, C.; Chen, Z.; Jin, Y.; Wang, Y.; Kolokythas, A.; Dai, Y.; Zhou, X. MicroRNA-138 suppresses epithelial-mesenchymal transition in squamous cell carcinoma cell lines. *Biochem J* **2011**, *440*, 23–31, doi:10.1042/BJ20111006.
12. Pandit, K. V.; Corcoran, D.; Yousef, H.; Yarlagadda, M.; Tzouvelekis, A.; Gibson, K. F.; Konishi, K.; Yousem, S. A.; Singh, M.; Handley, D.; Richards, T.; Selman, M.; Watkins, S. C.; Pardo, A.; Ben-Yehudah, A.; Bouros, D.; Eickelberg, O.; Ray, P.; Benos, P. V.; Kaminski, N. Inhibition and role of let-7d in idiopathic pulmonary fibrosis. *Am J Respir Crit Care Med* **2010**, *182*, 220–9, doi:10.1164/rccm.200911-1698OC.
13. Geraldo, M. V.; Yamashita, A. S.; Kimura, E. T. MicroRNA miR-146b-5p regulates signal transduction of TGF-beta by repressing SMAD4 in thyroid cancer. *Oncogene* **2012**, *31*, 1910–22, doi:10.1038/onc.2011.381.
14. Al-Ansari, M. M.; Aboussekhra, A. miR-146b-5p mediates p16-dependent repression of IL-6 and suppresses paracrine procarcinogenic effects of breast stromal fibroblasts. *Oncotarget* **2015**, *6*, 30006–16, doi:10.18632/oncotarget.4933.
15. Liu, Z.; Lu, C. L.; Cui, L. P.; Hu, Y. L.; Yu, Q.; Jiang, Y.; Ma, T.; Jiao, D. K.; Wang, D.; Jia, C. Y. MicroRNA-146a modulates TGF-beta1-induced phenotypic differentiation in human dermal fibroblasts by targeting SMAD4. *Arch Dermatol Res* **2012**, *304*, 195–202, doi:10.1007/s00403-011-1178-0.

16. He, Y.; Huang, C.; Sun, X.; Long, X. R.; Lv, X. W.; Li, J. MicroRNA-146a modulates TGF-beta1-induced hepatic stellate cell proliferation by targeting SMAD4. *Cell Signal* **2012**, *24*, 1923–30, doi:10.1016/j.cellsig.2012.06.003.
17. Morishita, Y.; Imai, T.; Yoshizawa, H.; Watanabe, M.; Ishibashi, K.; Muto, S.; Nagata, D. Delivery of microRNA-146a with polyethylenimine nanoparticles inhibits renal fibrosis in vivo. *Int J Nanomedicine* **2015**, *10*, 3475–88, doi:10.2147/IJN.S82587.
18. Pottier, N.; Maurin, T.; Chevalier, B.; Puissegur, M. P.; Lebrigand, K.; Robbe-Sermesant, K.; Bertero, T.; Lino Cardenas, C. L.; Courcot, E.; Rios, G.; Fourre, S.; Lo-Guidice, J. M.; Marcet, B.; Cardinaud, B.; Barbry, P.; Mari, B. Identification of keratinocyte growth factor as a target of microRNA-155 in lung fibroblasts: implication in epithelial-mesenchymal interactions. *PLoS One* **2009**, *4*, e6718, doi:10.1371/journal.pone.0006718.
19. Louafi, F.; Martinez-Nunez, R. T.; Sanchez-Elsner, T. MicroRNA-155 targets SMAD2 and modulates the response of macrophages to transforming growth factor-{beta}. *J Biol Chem* **2010**, *285*, 41328–36, doi:10.1074/jbc.M110.146852.
20. Kong, W.; Yang, H.; He, L.; Zhao, J. J.; Coppola, D.; Dalton, W. S.; Cheng, J. Q. MicroRNA-155 is regulated by the transforming growth factor beta/Smad pathway and contributes to epithelial cell plasticity by targeting RhoA. *Mol Cell Biol* **2008**, *28*, 6773–84, doi:10.1128/MCB.00941-08.
21. Gennarino, V. A.; D'Angelo, G.; Dharmalingam, G.; Fernandez, S.; Russolillo, G.; Sanges, R.; Mutarelli, M.; Belcastro, V.; Ballabio, A.; Verde, P.; Sardiello, M.; Banfi, S. Identification of microRNA-regulated gene networks by expression analysis of target genes. *Genome Res* **2012**, *22*, 1163–72, doi:10.1101/gr.130435.111.
22. Dakhlallah, D.; Batte, K.; Wang, Y.; Cantemir-Stone, C. Z.; Yan, P.; Nuovo, G.; Mikhail, A.; Hitchcock, C. L.; Wright, V. P.; Nana-Sinkam, S. P.; Piper, M. G.; Marsh, C. B. Epigenetic regulation of miR-17~92 contributes to the pathogenesis of pulmonary fibrosis. *Am J Respir Crit Care Med* **2013**, *187*, 397–405, doi:10.1164/rccm.201205-0888OC.
23. Lu, L.; Wang, J.; Lu, H.; Zhang, G.; Liu, Y.; Wang, J.; Zhang, Y.; Shang, H.; Ji, H.; Chen, X.; Duan, Y.; Li, Y. MicroRNA-130a and -130b enhance activation of hepatic stellate cells by suppressing PPARgamma expression: A rat fibrosis model study. *Biochem Biophys Res Commun* **2015**, *465*, 387–93, doi:10.1016/j.bbrc.2015.08.012.
24. Chen, Q. G.; Zhou, W.; Han, T.; Du, S. Q.; Li, Z. H.; Zhang, Z.; Shan, G. Y.; Kong, C. Z. MiR-345 suppresses proliferation, migration and invasion by targeting Smad1 in human prostate cancer. *J Cancer Res Clin Oncol* **2016**, *142*, 213–24, doi:10.1007/s00432-015-2016-0.
25. Petrocca, F.; Visone, R.; Onelli, M. R.; Shah, M. H.; Nicoloso, M. S.; de Martino, I.; Iliopoulos, D.; Pilozzi, E.; Liu, C. G.; Negrini, M.; Cavazzini, L.; Volinia, S.; Alder, H.; Ruco, L. P.; Baldassarre, G.; Croce, C. M.; Vecchione, A. E2F1-regulated microRNAs impair TGFbeta-dependent cell-cycle arrest and apoptosis in gastric cancer. *Cancer Cell* **2008**, *13*, 272–86, doi:10.1016/j.ccr.2008.02.013.
26. Smith, A. L.; Iwanaga, R.; Drasin, D. J.; Micalizzi, D. S.; Vartuli, R. L.; Tan, A. C.; Ford, H. L. The miR-106b-25 cluster targets Smad7, activates TGF-beta signaling, and induces EMT and tumor initiating cell characteristics downstream of Six1 in human breast cancer. *Oncogene* **2012**, *31*, 5162–71, doi:10.1038/onc.2012.11.
27. Qu, M. H.; Han, C.; Srivastava, A. K.; Cui, T.; Zou, N.; Gao, Z. Q.; Wang, Q. E. miR-93 promotes TGF-beta-induced epithelial-to-mesenchymal transition through downregulation of NEDD4L in lung cancer cells. *Tumour Biol* **2015**, doi:10.1007/s13277-015-4328-8.
28. Lyu, X.; Fang, W.; Cai, L.; Zheng, H.; Ye, Y.; Zhang, L.; Li, J.; Peng, H.; Cho, W. C.; Wang, E.; Marincola, F. M.; Yao, K.; Cai, H.; Li, J.; Li, X. TGFbetaR2 is a major target of miR-93 in nasopharyngeal carcinoma aggressiveness. *Mol Cancer* **2014**, *13*, 51, doi:10.1186/1476-4598-13-51.

29. Oak, S. R.; Murray, L.; Herath, A.; Sleeman, M.; Anderson, I.; Joshi, A. D.; Coelho, A. L.; Flaherty, K. R.; Toews, G. B.; Knight, D.; Martinez, F. J.; Hogaboam, C. M. A micro RNA processing defect in rapidly progressing idiopathic pulmonary fibrosis. *PLoS One* **2011**, *6*, e21253, doi:10.1371/journal.pone.0021253.
30. Chen, C.; Wu, C. Q.; Zhang, Z. Q.; Yao, D. K.; Zhu, L. Loss of expression of miR-335 is implicated in hepatic stellate cell migration and activation. *Exp Cell Res* **2011**, *317*, 1714–25, doi:10.1016/j.yexcr.2011.05.001.
31. Zhang, J.; Tu, Q.; Bonewald, L. F.; He, X.; Stein, G.; Lian, J.; Chen, J. Effects of miR-335-5p in modulating osteogenic differentiation by specifically downregulating Wnt antagonist DKK1. *J Bone Min. Res* **2011**, *26*, 1953–63, doi:10.1002/jbmr.377.
32. Lin, X.; Wu, L.; Zhang, Z.; Yang, R.; Guan, Q.; Hou, X.; Wu, Q. MiR-335-5p promotes chondrogenesis in mouse mesenchymal stem cells and is regulated through two positive feedback loops. *J Bone Min. Res* **2014**, *29*, 1575–85, doi:10.1002/jbmr.2163.
33. Milosevic, J.; Pandit, K.; Magister, M.; Rabinovich, E.; Ellwanger, D. C.; Yu, G.; Vuga, L. J.; Weksler, B.; Benos, P. V.; Gibson, K. F.; McMillan, M.; Kahn, M.; Kaminski, N. Profibrotic role of miR-154 in pulmonary fibrosis. *Am J Respir Cell Mol Biol* **2012**, *47*, 879–87, doi:10.1165/rcmb.2011-0377OC.
34. He, Y.; Huang, C.; Lin, X.; Li, J. MicroRNA-29 family, a crucial therapeutic target for fibrosis diseases. *Biochimie* **2013**, *95*, 1355–9, doi:10.1016/j.biochi.2013.03.010.
35. Montgomery, R. L.; Yu, G.; Latimer, P. A.; Stack, C.; Robinson, K.; Dalby, C. M.; Kaminski, N.; van Rooij, E. MicroRNA mimicry blocks pulmonary fibrosis. *EMBO Mol Med* **2014**, *6*, 1347–56, doi:10.15252/emmm.201303604.
36. Xiao, J.; Meng, X. M.; Huang, X. R.; Chung, A. C.; Feng, Y. L.; Hui, D. S.; Yu, C. M.; Sung, J. J.; Lan, H. Y. miR-29 inhibits bleomycin-induced pulmonary fibrosis in mice. *Mol Ther* **2012**, *20*, 1251–60, doi:10.1038/mt.2012.36.
37. Cushing, L.; Kuang, P. P.; Qian, J.; Shao, F.; Wu, J.; Little, F.; Thannickal, V. J.; Cardoso, W. V.; Lu, J. miR-29 is a major regulator of genes associated with pulmonary fibrosis. *Am J Respir Cell Mol Biol* **2011**, *45*, 287–94, doi:10.1165/rcmb.2010-0323OC.
38. Qin, W.; Chung, A. C.; Huang, X. R.; Meng, X. M.; Hui, D. S.; Yu, C. M.; Sung, J. J.; Lan, H. Y. TGF-beta/Smad3 signaling promotes renal fibrosis by inhibiting miR-29. *J Am Soc Nephrol* **2011**, *22*, 1462–74, doi:10.1681/ASN.2010121308.
39. Zhang, Y.; Huang, X. R.; Wei, L. H.; Chung, A. C.; Yu, C. M.; Lan, H. Y. miR-29b as a therapeutic agent for angiotensin II-induced cardiac fibrosis by targeting TGF-beta/Smad3 signaling. *Mol Ther* **2014**, *22*, 974–85, doi:10.1038/mt.2014.25.
40. van Rooij, E.; Sutherland, L. B.; Thatcher, J. E.; DiMaio, J. M.; Naseem, R. H.; Marshall, W. S.; Hill, J. A.; Olson, E. N. Dysregulation of microRNAs after myocardial infarction reveals a role of miR-29 in cardiac fibrosis. *Proc Natl Acad Sci U A* **2008**, *105*, 13027–32, doi:10.1073/pnas.0805038105.
41. Roderburg, C.; Urban, G. W.; Bettermann, K.; Vucur, M.; Zimmermann, H.; Schmidt, S.; Janssen, J.; Koppe, C.; Knolle, P.; Castoldi, M.; Tacke, F.; Trautwein, C.; Luedde, T. Micro-RNA profiling reveals a role for miR-29 in human and murine liver fibrosis. *Hepatology* **2011**, *53*, 209–18, doi:10.1002/hep.23922.
42. Sekiya, Y.; Ogawa, T.; Yoshizato, K.; Ikeda, K.; Kawada, N. Suppression of hepatic stellate cell activation by microRNA-29b. *Biochem Biophys Res Commun* **2011**, *412*, 74–9, doi:10.1016/j.bbrc.2011.07.041.
43. Zhang, Y.; Wu, L.; Wang, Y.; Zhang, M.; Li, L.; Zhu, D.; Li, X.; Gu, H.; Zhang, C. Y.; Zen, K. Protective role of estrogen-induced miRNA-29 expression in carbon tetrachloride-induced mouse liver injury. *J Biol Chem* **2012**, *287*, 14851–62, doi:10.1074/jbc.M111.314922.
44. Maurer, B.; Stanczyk, J.; Jungel, A.; Akhmetshina, A.; Trenkmann, M.; Brock, M.; Kowal-Bielecka, O.; Gay, R. E.; Michel, B. A.; Distler, J. H.; Gay, S.; Distler, O. MicroRNA-29, a key regulator of collagen expression in systemic sclerosis. *Arthritis Rheum* **2010**, *62*, 1733–43, doi:10.1002/art.27443.

45. Cushing, L.; Kuang, P.; Lu, J. The role of miR-29 in pulmonary fibrosis. *Biochem Cell Biol* **2015**, *93*, 109–18, doi:10.1139/bcb-2014-0095.

46. Pandit, K. V.; Milosevic, J. MicroRNA regulatory networks in idiopathic pulmonary fibrosis. *Biochem Cell Biol* **2015**, *93*, 129–37, doi:10.1139/bcb-2014-0101.

47. Zhao, S.; Zhang, Y.; Zheng, X.; Tu, X.; Li, H.; Chen, J.; Zang, Y.; Zhang, J. Loss of MicroRNA-101 Promotes Epithelial to Mesenchymal Transition in Hepatocytes. *J Cell Physiol* **2015**, *230*, 2706–17, doi:10.1002/jcp.24995.

48. Tu, X.; Zhang, H.; Zhang, J.; Zhao, S.; Zheng, X.; Zhang, Z.; Zhu, J.; Chen, J.; Dong, L.; Zang, Y.; Zhang, J. MicroRNA-101 suppresses liver fibrosis by targeting the TGFbeta signalling pathway. *J Pathol* **2014**, *234*, 46–59, doi:10.1002/path.4373.

49. Li, P.; Li, J.; Chen, T.; Wang, H.; Chu, H.; Chang, J.; Zang, W.; Wang, Y.; Ma, Y.; Du, Y.; Zhao, G.; Zhang, G. Expression analysis of serum microRNAs in idiopathic pulmonary fibrosis. *Int J Mol Med* **2014**, *33*, 1554–62, doi:10.3892/ijmm.2014.1712.

50. Pan, Z.; Sun, X.; Shan, H.; Wang, N.; Wang, J.; Ren, J.; Feng, S.; Xie, L.; Lu, C.; Yuan, Y.; Zhang, Y.; Wang, Y.; Lu, Y.; Yang, B. MicroRNA-101 inhibited postinfarct cardiac fibrosis and improved left ventricular compliance via the FBJ osteosarcoma oncogene/transforming growth factor-beta1 pathway. *Circulation* **2012**, *126*, 840–50, doi:10.1161/CIRCULATIONAHA.112.094524.

51. Murakami, Y.; Toyoda, H.; Tanaka, M.; Kuroda, M.; Harada, Y.; Matsuda, F.; Tajima, A.; Kosaka, N.; Ochiya, T.; Shimotohno, K. The progression of liver fibrosis is related with overexpression of the miR-199 and 200 families. *PLoS One* **2011**, *6*, e16081, doi:10.1371/journal.pone.0016081.

52. Lino Cardenas, C. L.; Henaoui, I. S.; Courcot, E.; Roderburg, C.; Cauffiez, C.; Aubert, S.; Copin, M. C.; Wallaert, B.; Glowacki, F.; Dewaeles, E.; Milosevic, J.; Maurizio, J.; Tedrow, J.; Marcet, B.; Lo-Guidice, J. M.; Kaminski, N.; Barbry, P.; Luedde, T.; Perrais, M.; Mari, B.; Pottier, N. miR-199a-5p Is upregulated during fibrogenic response to tissue injury and mediates TGFbeta-induced lung fibroblast activation by targeting caveolin-1. *PLoS Genet* **2013**, *9*, e1003291, doi:10.1371/journal.pgen.1003291.

53. Ji, X.; Wu, B.; Fan, J.; Han, R.; Luo, C.; Wang, T.; Yang, J.; Han, L.; Zhu, B.; Wei, D.; Chen, J.; Ni, C. The Anti-fibrotic Effects and Mechanisms of MicroRNA-486-5p in Pulmonary Fibrosis. *Sci Rep* **2015**, *5*, 14131, doi:10.1038/srep14131.

54. Lu, C. H.; Hou, Q. R.; Deng, L. F.; Fei, C.; Xu, W. P.; Zhang, Q.; Wu, K. M.; Ning, B. F.; Xie, W. F.; Zhang, X. MicroRNA-370 Attenuates Hepatic Fibrogenesis by Targeting Smoothened. *Dig Sci* **2015**, *60*, 2038–48, doi:10.1007/s10620-015-3585-0.
